# Supplementary material for: Individual differences in how infants change behaviours from spontaneous to instrumental
Source: Commun Psychol. 2025 Nov 20;3:161. doi: 10.1038/s44271-025-00333-3 (PMC12635129; doi:10.1038/s44271-025-00333-3)
Supplement: Supplementary file 2 — Supplementary Information [file 44271_2025_333_MOESM2_ESM.pdf]

**Supplemental Information**

# Individual differences in how infants change behaviours from spontaneous to instrumental

Ryo Fujihira, Hama Watanabe, and Gentaro Taga

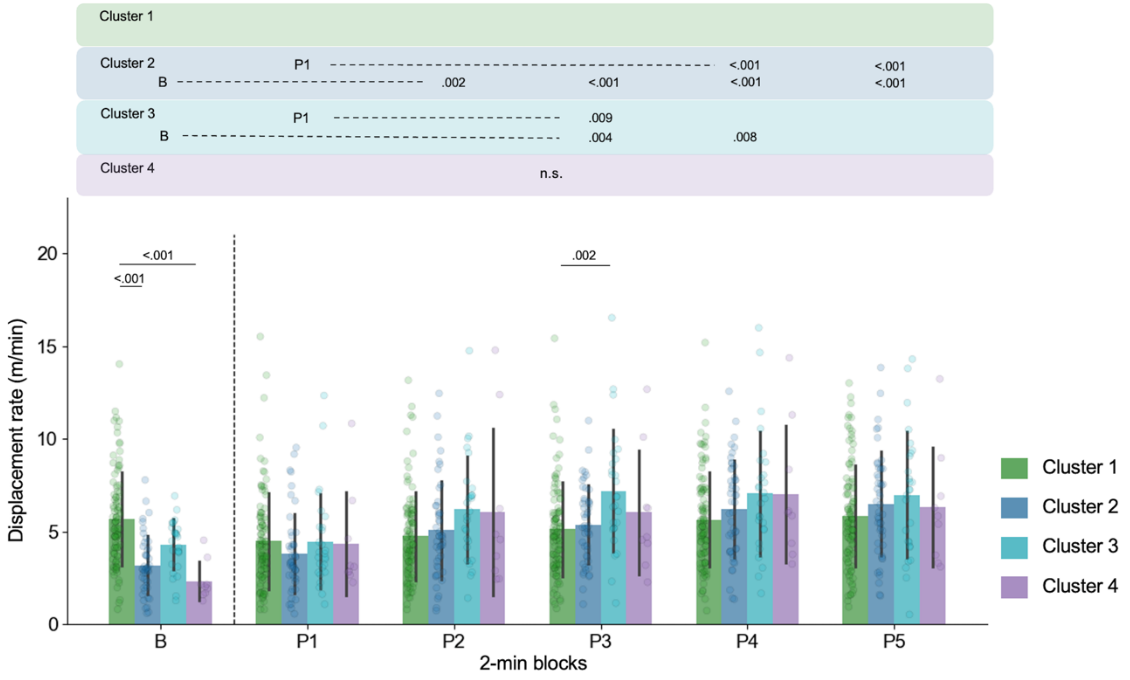


**Figure S1. Cluster-wise mean displacement rate of the unconnected arm**

Mean displacement rates in each phase are shown (B: baseline phase, P: play phase; P1: 0-2 min, P2: 2-4 min, P3: 4-6 min, P4: 6-8 min, P5: 8-10min). Error bars show the SD of the displacement rates in each cluster. Significant main effects were observed for the phases ($F$(5, 905) = 44.91, $p$ < 0.001, $\eta_p^2$ = 0.199, 95% CI = [0.152, 0.241]), but not for the clusters ($F$(3, 181) = 0.87, $p$ = 0.460, $\eta_p^2$ = 0.014, 95% CI = [0.0, 0.051]). In addition, significant interaction between the phases and clusters was observed ($F$(5, 905) = 17.24, $p$ < 0.001, $\eta_p^2$ = 0.087, 95% CI = [0.052, 0.120]). With regard to the interaction, there were significant simple main effects of the phases in cluster 2 ($F$(5, 905) = 15.84, $p$ < 0.001, $\eta_p^2$ = 0.080, 95% CI = [0.046, 0.112]), cluster 3 ($F$(5, 905) = 16.12, $p$ < 0.001, $\eta_p^2$ = 0.082, 95% CI = [0.047, 0.114]), and cluster 4 ($F$(5, 905) = 27.34, $p$ < 0.001, $\eta_p^2$ = 0.131, 95% CI = [0.090, 0.169]). With respect to the effect of clusters, there was a significant simple main effect of the clusters in baseline ($F$(3, 181) = 31.31, $p$ < 0.001, $\eta_p^2$ = 0.342, 95% CI = [0.230, 0.434]) and play 3 ($F$(3, 181) = 8.34, $p$ < 0.001, $\eta_p^2$ = 0.121, 95% CI = [0.039, 0.206]). The results of Tukey's HSD test were described in this figure and Supplementary Table 19 to 28. n.s. means that there was no statistical significance for multiple comparisons. We did not conduct the multiple comparisons in cluster 1, P1, P2, P4, and P5 because simple main effects of phases or clusters were not significant.

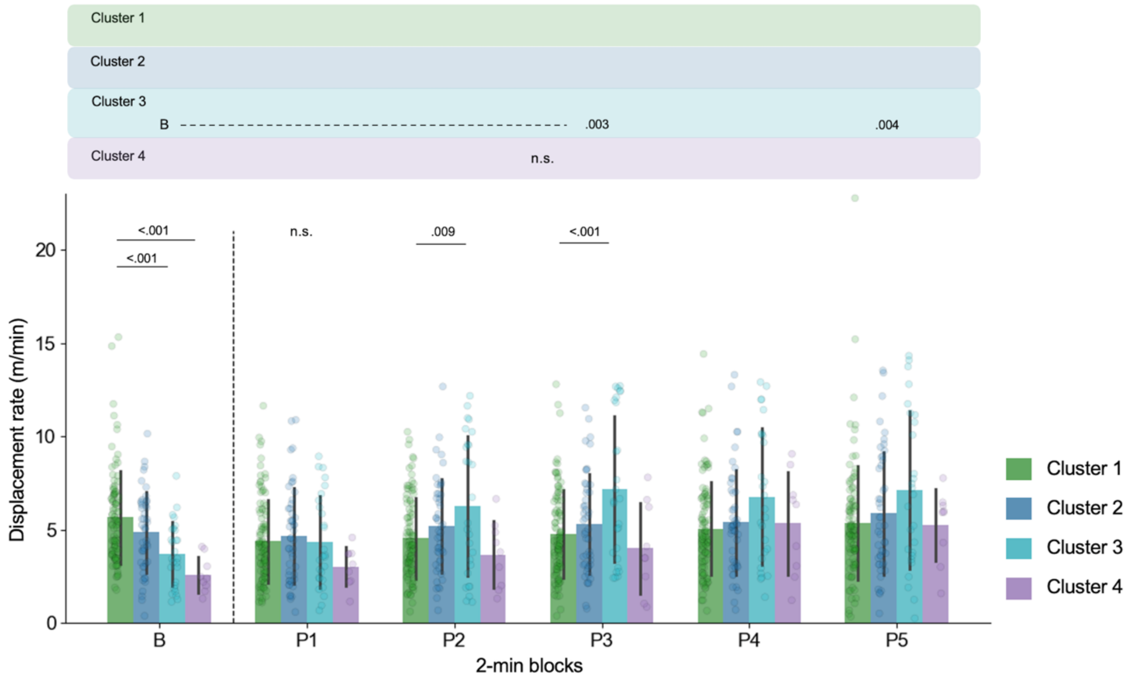


**Figure S2. Cluster-wise mean displacement rate of the ipsilateral leg**

Mean displacement rates in each phase are shown (B: baseline phase, P: play phase; P1: 0-2 min, P2: 2-4 min, P3: 4-6 min, P4: 6-8 min, P5: 8-10min). Error bars show the SD of the displacement rates in each cluster. Significant main effects were observed for the phases ($F$(5, 905) =21.40, $p$ < 0.001, $\eta_p^2$ = 0.106, 95% CI = [0.068, 0.141]), but not for the clusters ($F$(3, 181) = 2.65, $p$ = 0.051, $\eta_p^2$ = 0.042, 95% CI = [0.0, 0.101]). In addition, significant interaction between the phases and clusters was observed ($F$(5, 905) = 17.08, $p$ < 0.001, $\eta_p^2$ = 0.086, 95% CI = [0.051, 0.119]). With regard to the interaction, there were significant simple main effects of the phases in cluster 3 ($F$(5, 905) = 21.84, $p$ < 0.001, $\eta_p^2$ = 0.108, 95% CI = [0.069, 0.143]) and cluster 4 ($F$(5, 905) = 12.58, $p$ < 0.001, $\eta_p^2$ = 0.065, 95% CI = [0.034, 0.094]). With respect to the effect of clusters, there was a significant simple main effect of the clusters in baseline ($F$(3, 181) = 23.63, $p$ < 0.001, $\eta_p^2$ = 0.281, 95% CI = [0.172, 0.376]), play 1 ($F$(3, 181) = 6.64, $p$ < 0.001, $\eta_p^2$ = 0.099, 95% CI = [0.024, 0.179]), play 2 ($F$(3, 181) = 12.42, $p$ < 0.001, $\eta_p^2$ = 0.171, 95% CI = [0.075, 0.262]), and play 3 ($F$(3, 181) = 16.36, $p$ < 0.001, $\eta_p^2$ = 0.213, 95% CI = [0.110, 0.307]). The results of Tukey's HSD test were described in this figure and Supplementary Table 29 to 38. n.s. means that there was no statistical significance for multiple comparisons. We did not conduct the multiple comparisons in cluster 1, cluster 2, P4, and P5 because simple main effects of phases or clusters were not significant.

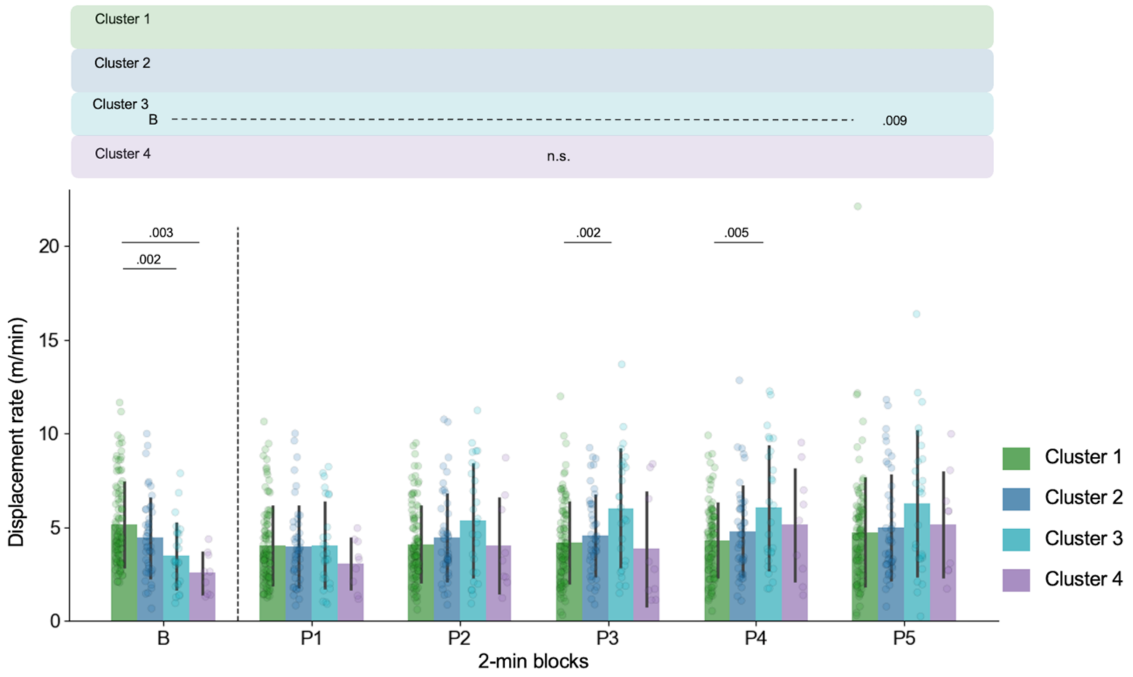


**Figure S3. Cluster-wise mean displacement rate of the contralateral leg**

Mean displacement rates in each phase are shown (B: baseline phase, P: play phase; P1: 0-2 min, P2: 2-4 min, P3: 4-6 min, P4: 6-8 min, P5: 8-10min). Error bars show the SD of the displacement rates in each cluster. Significant main effects were observed for the phases ($F$(5, 905) =17.78, $p$ < 0.001, $\eta_p^2$ = 0.089, 95% CI = [0.054, 0.122]), but not for the clusters ($F$(3, 181) =1.37, $p$ = 0.254, $\eta_p^2$ = 0.022, 95% CI = [0.0, 0.068]). In addition, significant interaction between the phases and clusters was observed ($F$(5, 905) = 16.09, $p$ < 0.001, $\eta_p^2$ = 0.082, 95% CI = [0.047, 0.114]). With regard to the interaction, there were significant simple main effects of the phases in cluster 3 ($F$(5, 905) = 16.67, $p$ < 0.001, $\eta_p^2$ = 0.084, 95% CI = [0.050, 0.117]) and cluster 4 ($F$(5, 905) = 13.47, $p$ < 0.001, $\eta_p^2$ = 0.069, 95% CI = [0.037, 0.099]). With respect to the effect of clusters, there was a significant simple main effect of the clusters in baseline ($F$(3, 181) = 19.05, $p$ < 0.001, $\eta_p^2$ = 0.240, 95% CI = [0.133, 0.335]), play 3 ($F$(3, 181) = 11.00, $p$ < 0.001, $\eta_p^2$ = 0.154, 95% CI = [0.062, 0.244]), and play 4 ($F$(3, 181) = 6.27, $p$ < 0.001, $\eta_p^2$ = 0.094, 95% CI = [0.021, 0.173]). The results of Tukey's HSD test were described in this figure and Supplementary Table 39 to 48. n.s. means that there was no statistical significance for multiple comparisons. We did not conduct the multiple comparisons in cluster 1, cluster 2, P1, P2, and P5 because simple main effects of phases were not significant.

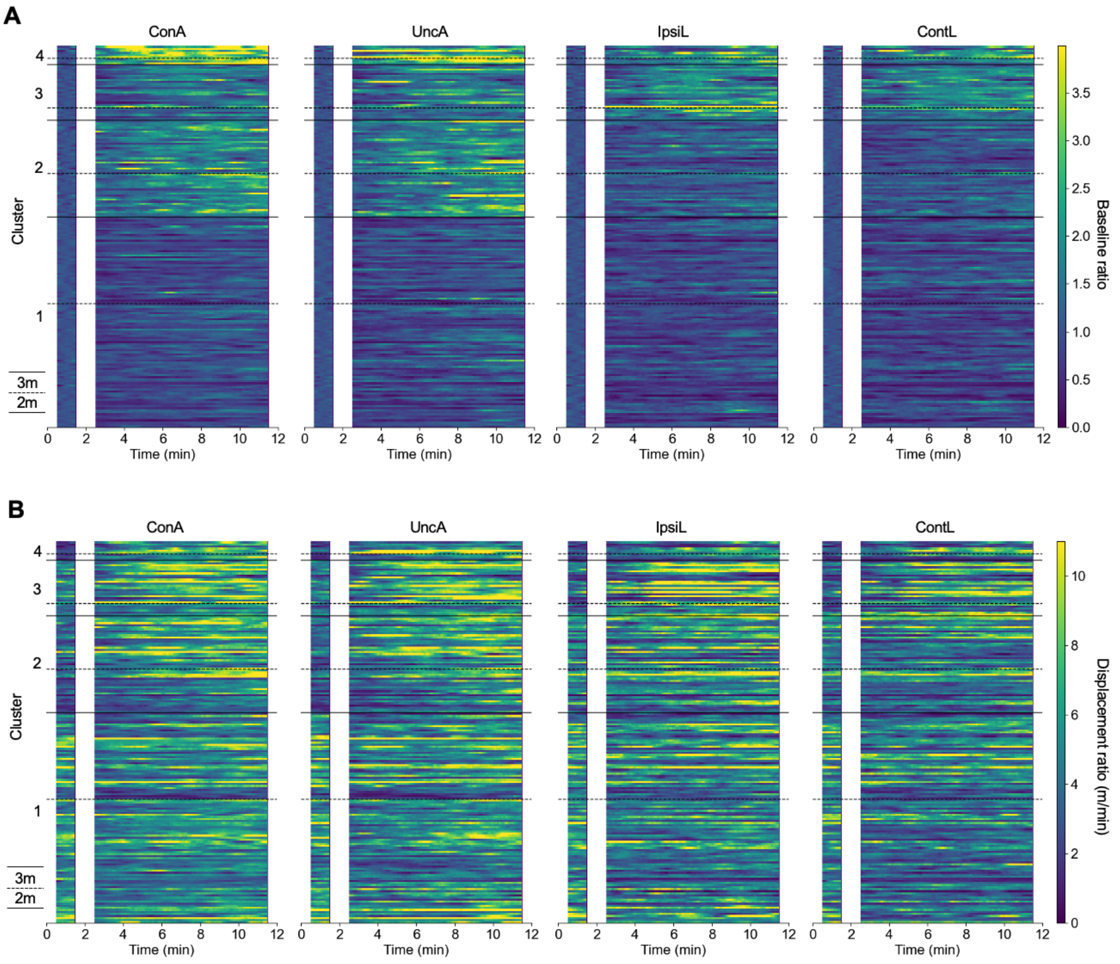


**Figure S4. Infant-wise clustered time evolutions in limb movements**

(A) The time evolutions in baseline ratio. Solid line divides clusters and dashed line divides 2- or 3-month-old infants within a cluster. ConA: connected arm, UncA: unconnected arm, IpsiL: ipsilateral leg, ContL: contralateral leg.

(B) The time evolutions in displacement rate.

## Cluster 1

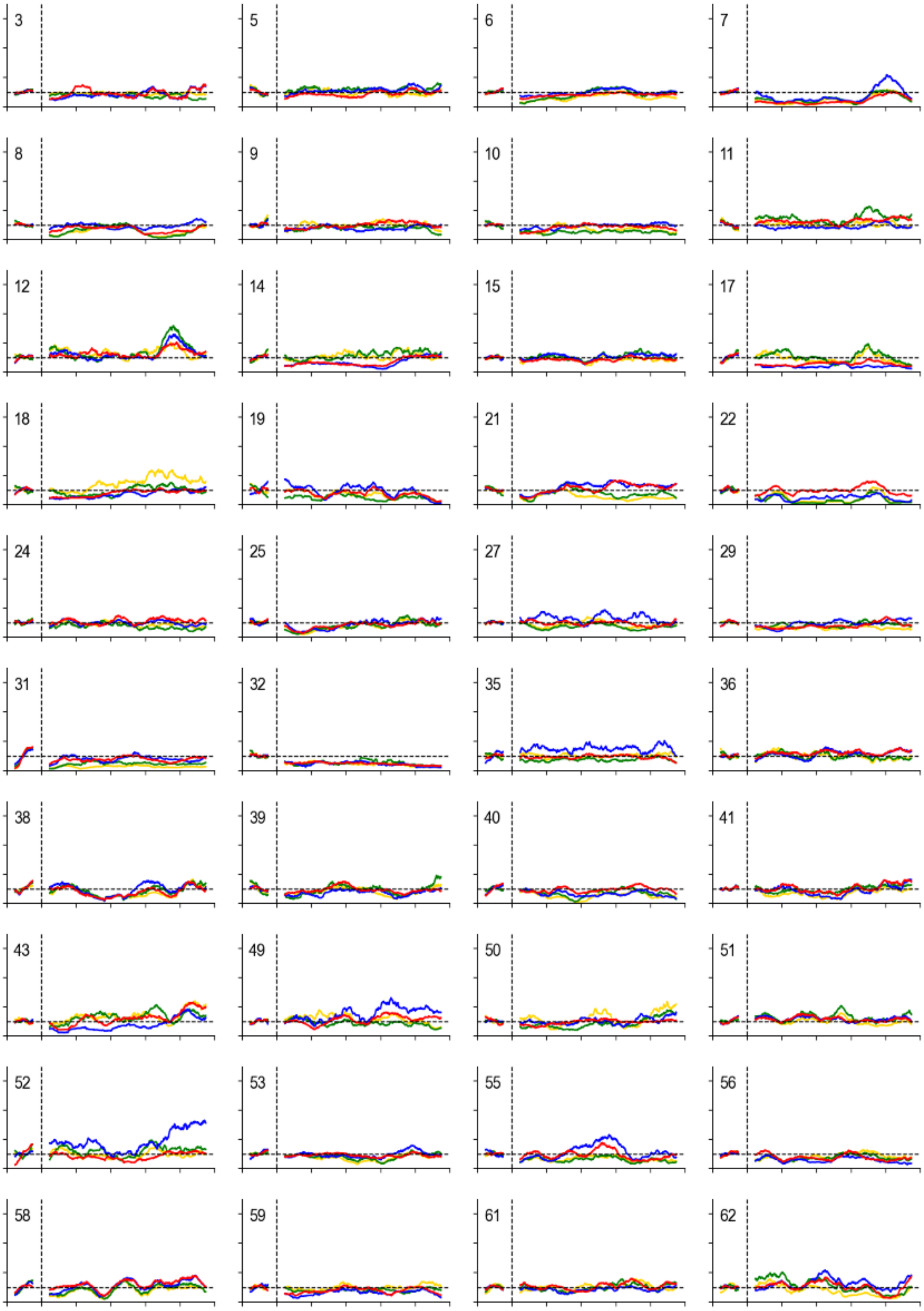
3
5
6
7
8
9
10
11
12
14
15
17
18
19
21
22
24
25
27
29
31
32
35
36
38
39
40
41
43
49
50
51
52
53
55
56
58
59
61
62

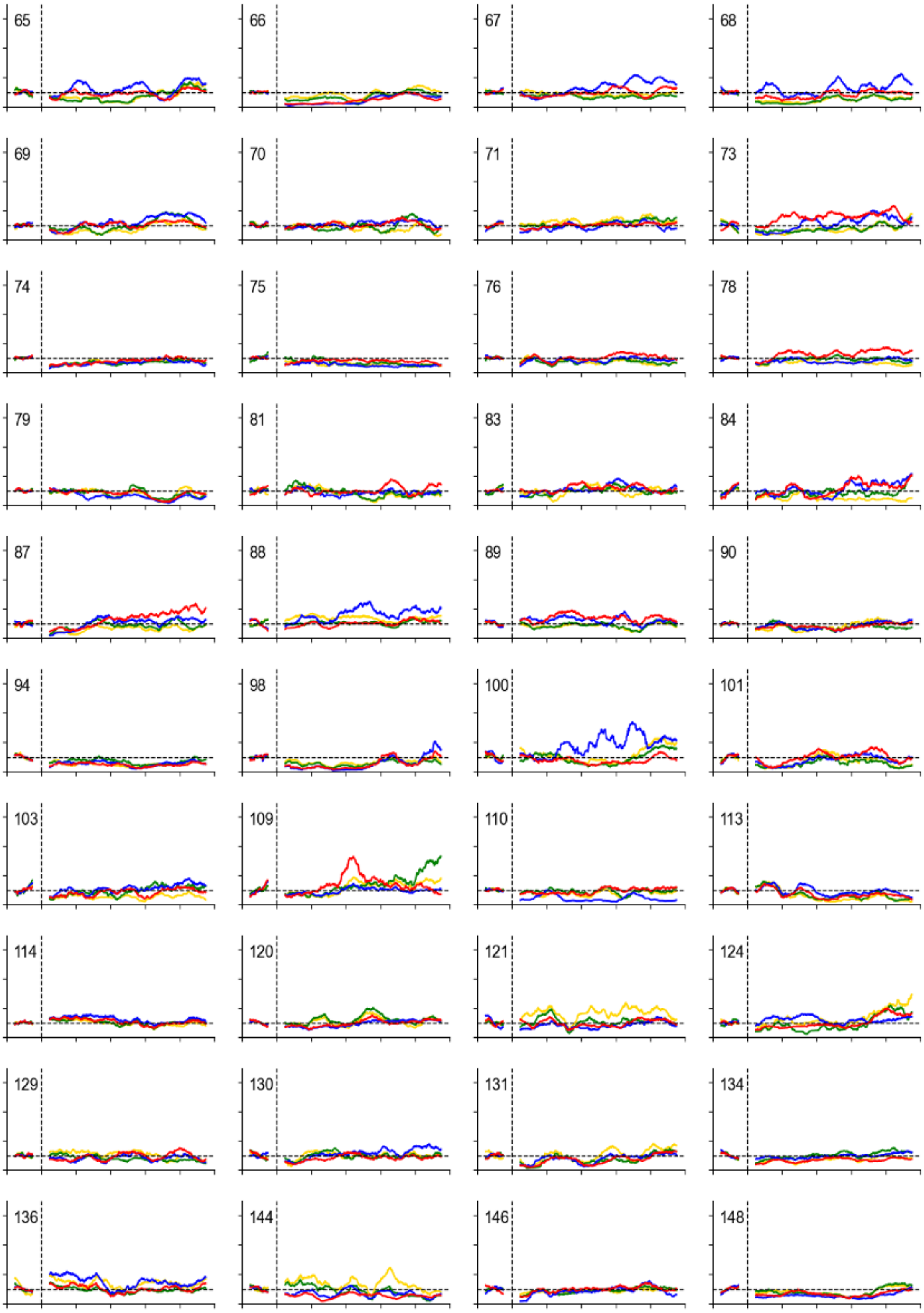
65
66
67
68
69
70
71
73
74
75
76
78
79
81
83
84
87
88
89
90
94
98
100
101
103
109
110
113
114
120
121
124
129
130
131
134
136
144
146
148

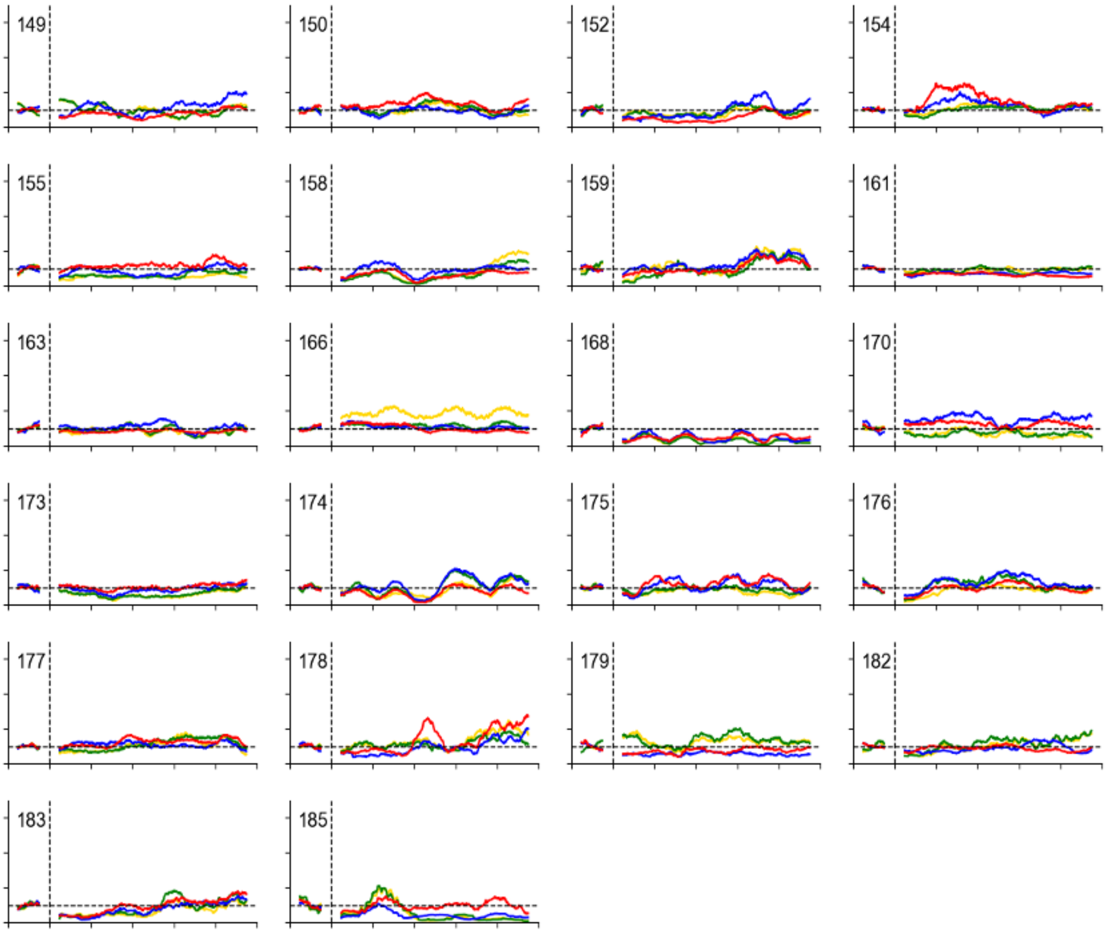
149
150
152
154
155
158
159
161
163
166
168
170
173
174
175
176
177
178
179
182
183
185

## Cluster 2

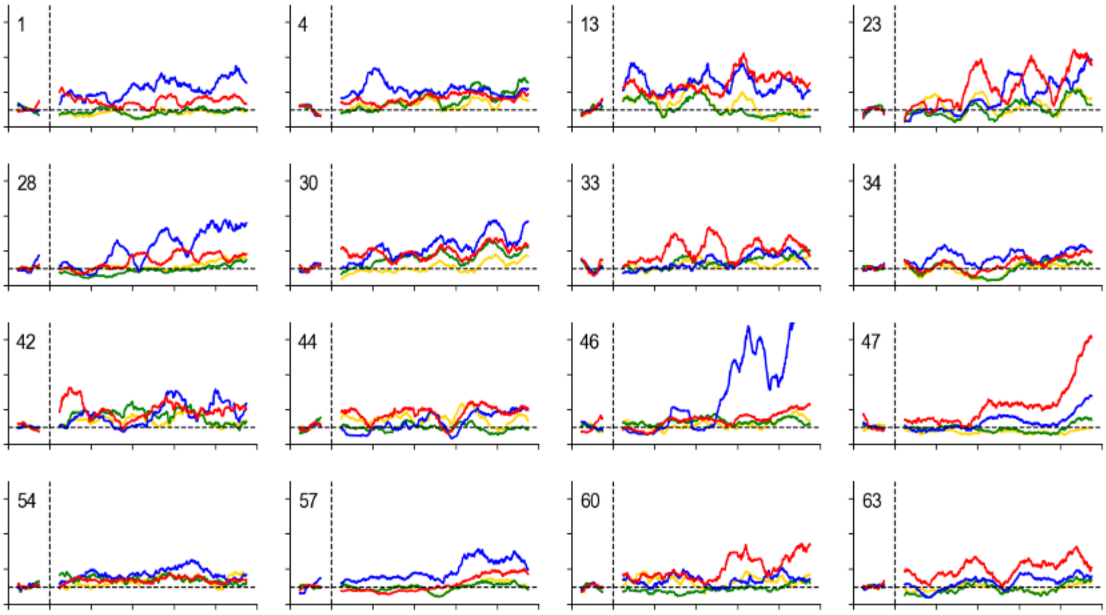
1
4
13
23
28
30
33
34
42
44
46
47
54
57
60
63

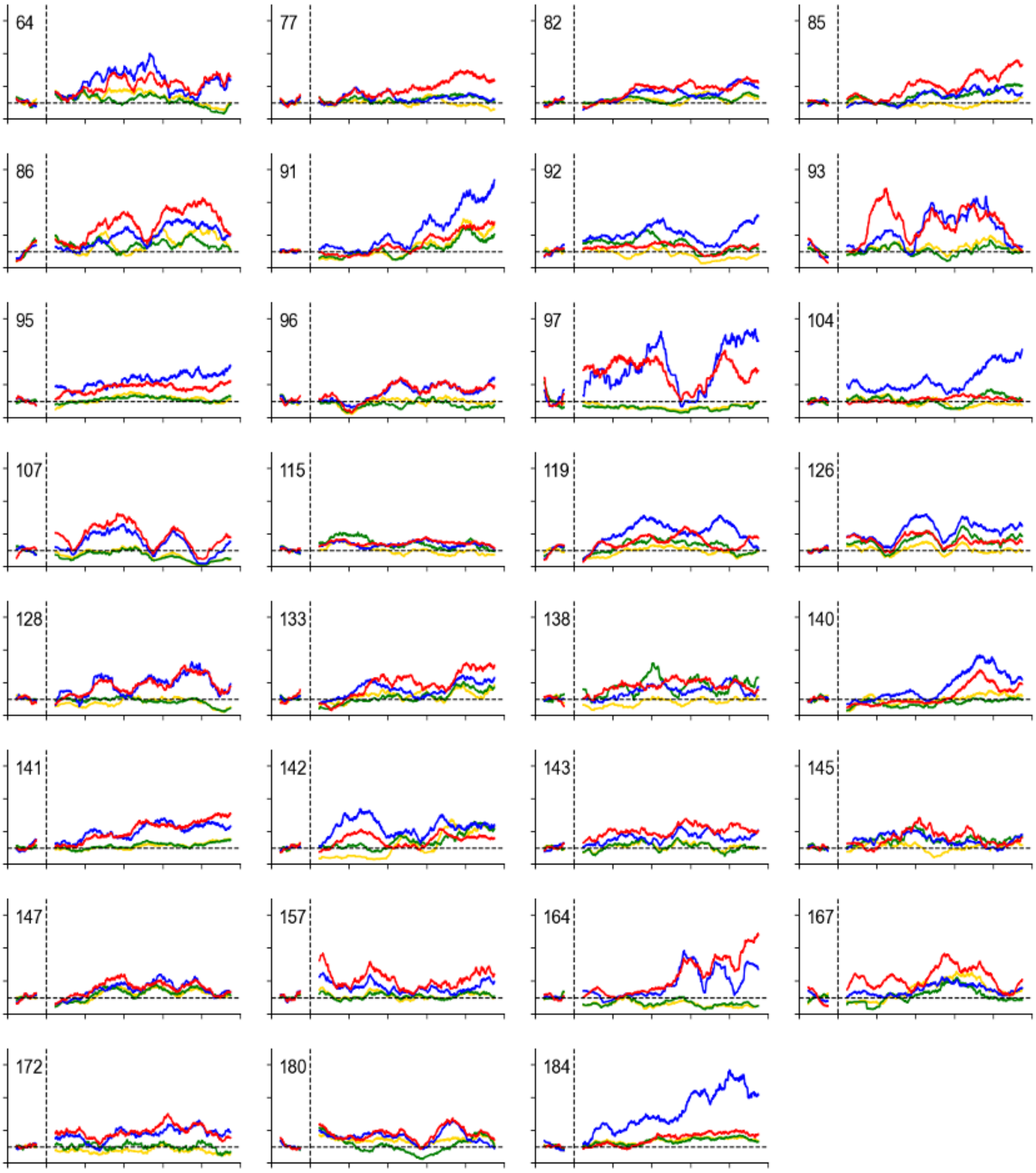
64
77
82
85
86
91
92
93
95
96
97
104
107
115
119
126
128
133
138
140
141
142
143
145
147
157
164
167
172
180
184

## Cluster 3

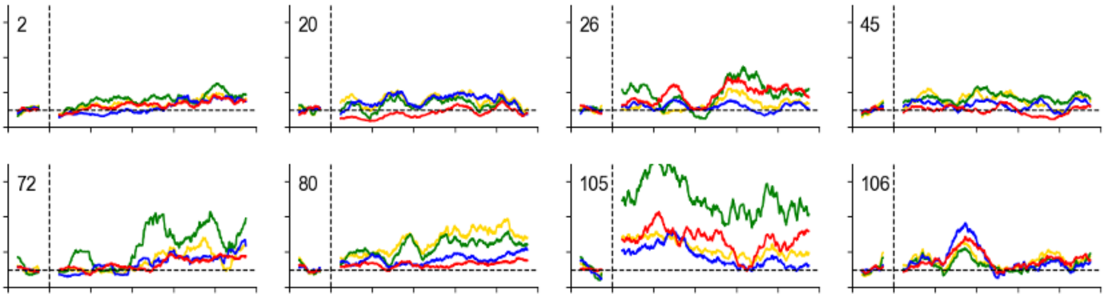
2
20
26
45
72
80
105
106

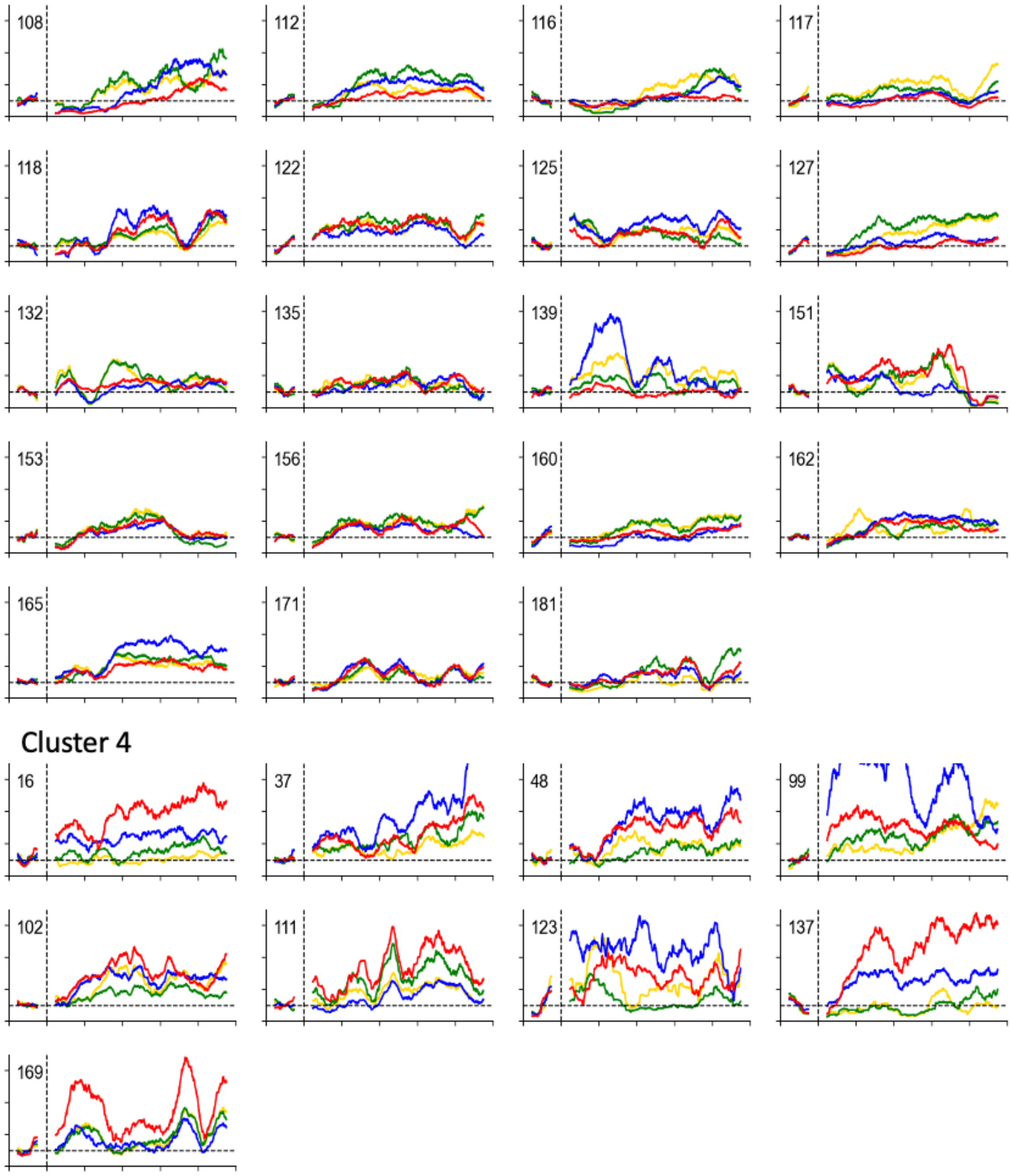


**Figure S5. Infant-wise time evolutions of baseline ratio for each limb**

ConA (red), UncA (blue), IpsiL (green), ContL (yellow). Panels are divided by the clusters of baseline ratio (main Figure 5 and 6). The number written in each panel is infant's ID. 2-month-old infants are from 1 to 90, and 3-month-old infants are from 91 to 185. The maximum value of Y-axis is 7. Though baseline ratios of some infants exceeded 7, this scale was most plausible to exhibit all time evolutions.

## Cluster 1

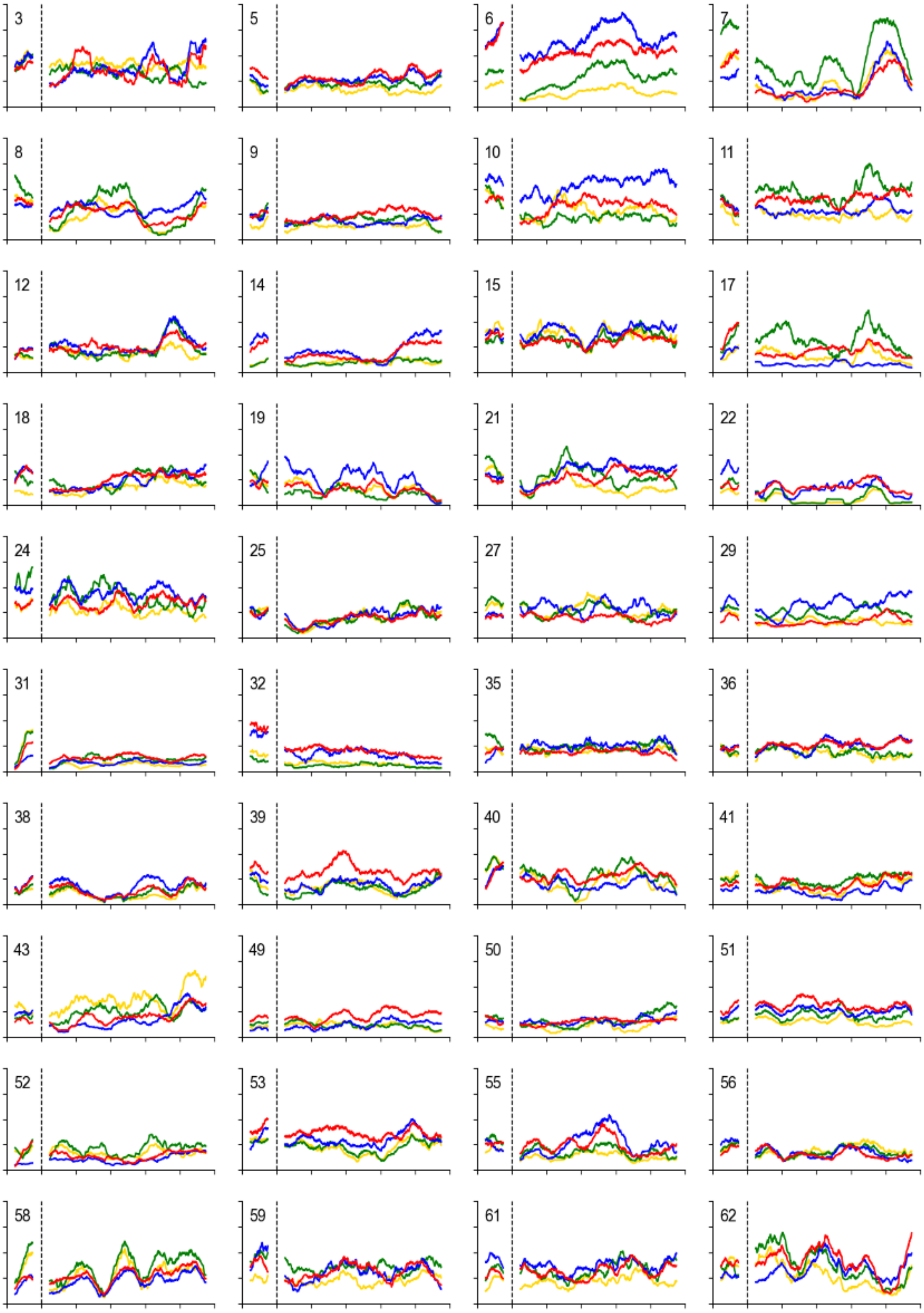
3
5
6
7
8
9
10
11
12
14
15
17
18
19
21
22
24
25
27
29
31
32
35
36
38
39
40
41
43
49
50
51
52
53
55
56
58
59
61
62

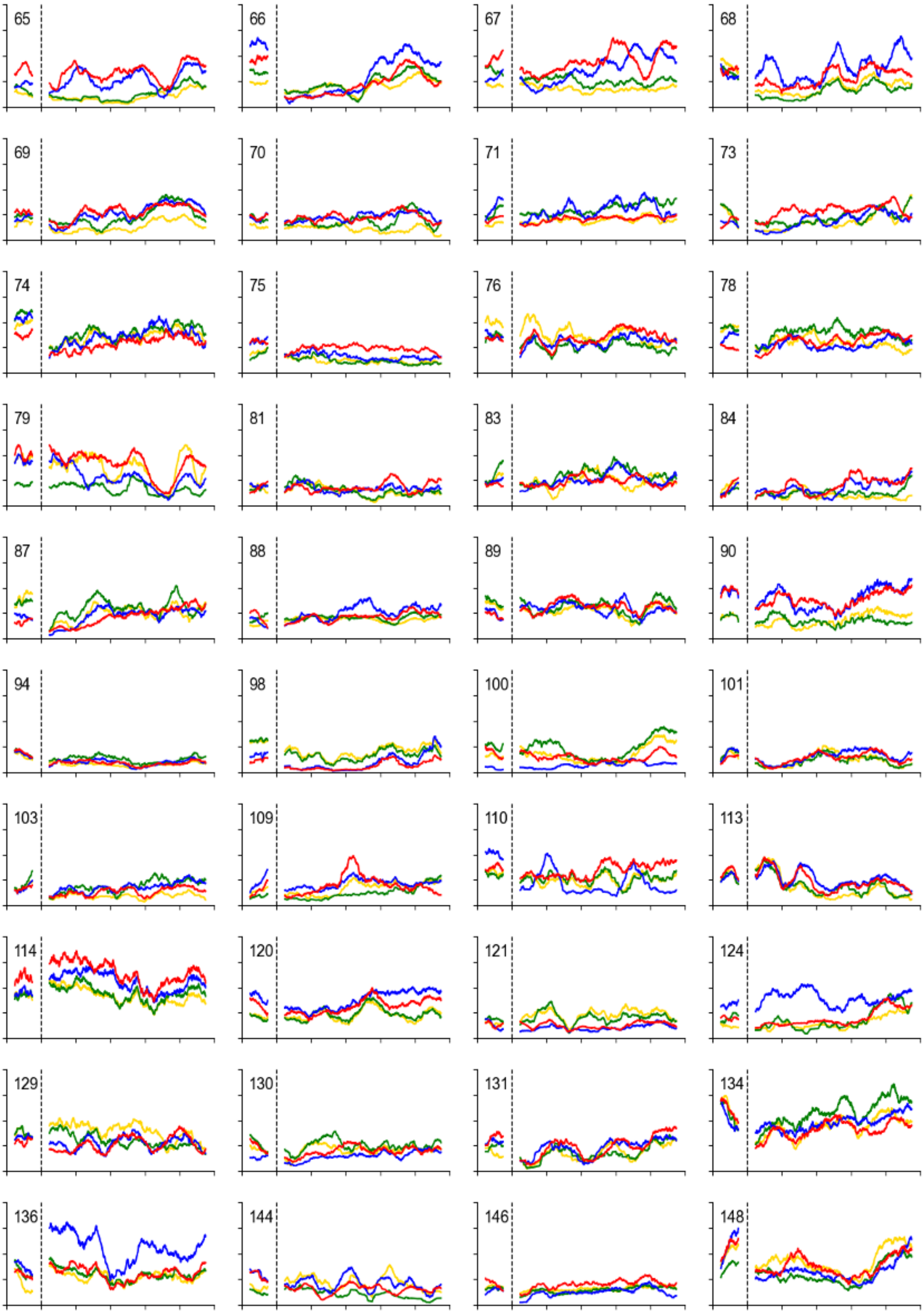
65
66
67
68
69
70
71
73
74
75
76
78
79
81
83
84
87
88
89
90
94
98
100
101
103
109
110
113
114
120
121
124
129
130
131
134
136
144
146
148

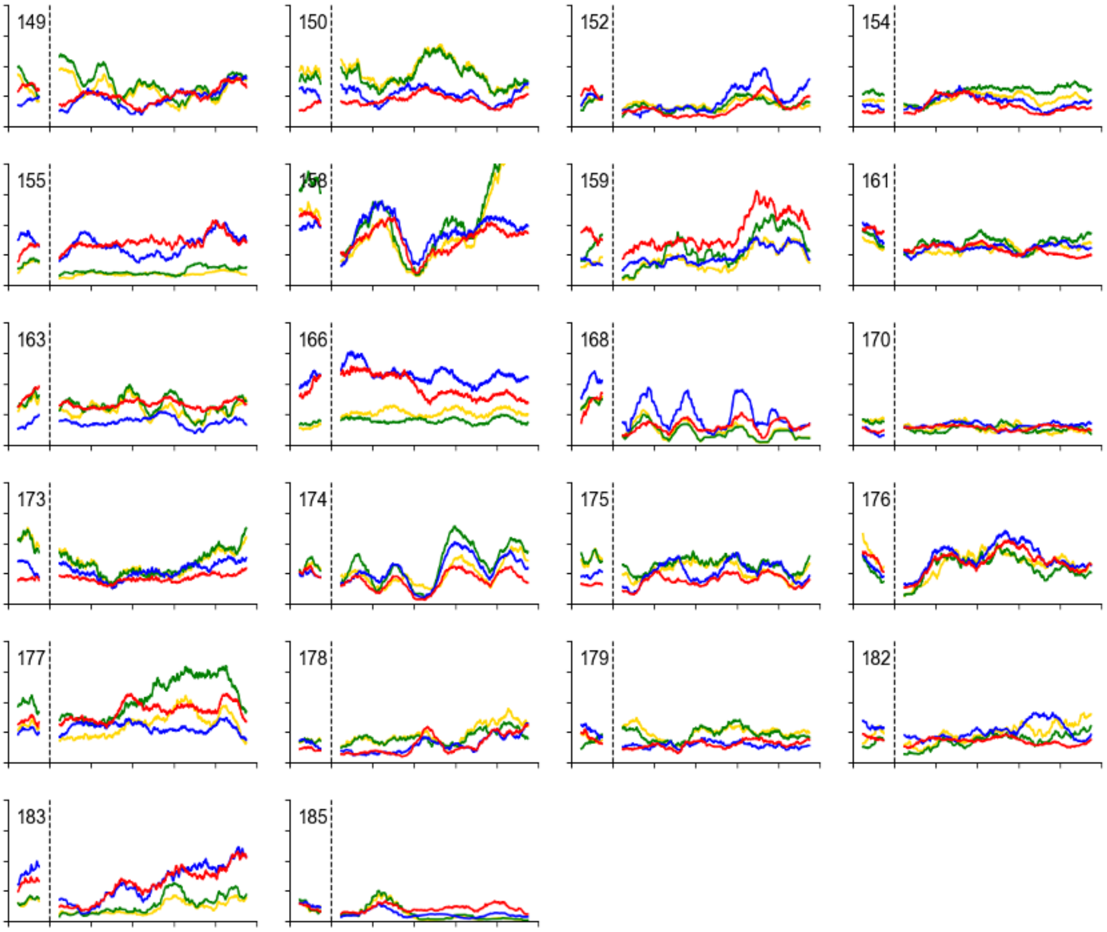
149
150
152
154
155
158
159
161
163
166
168
170
173
174
175
176
177
178
179
182
183
185

## Cluster 2

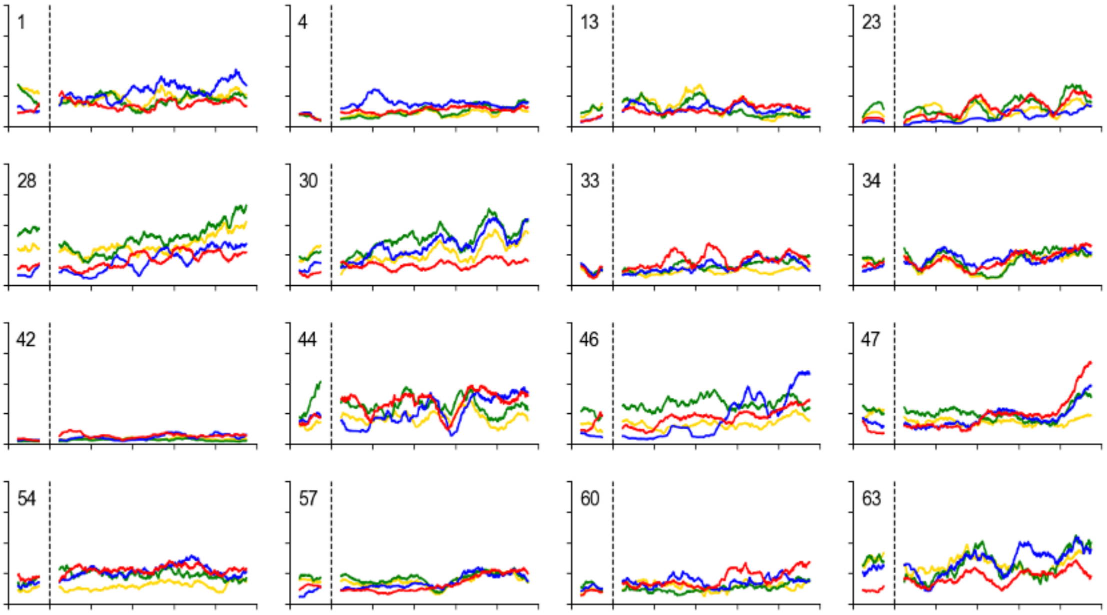
1
4
13
23
28
30
33
34
42
44
46
47
54
57
60
63

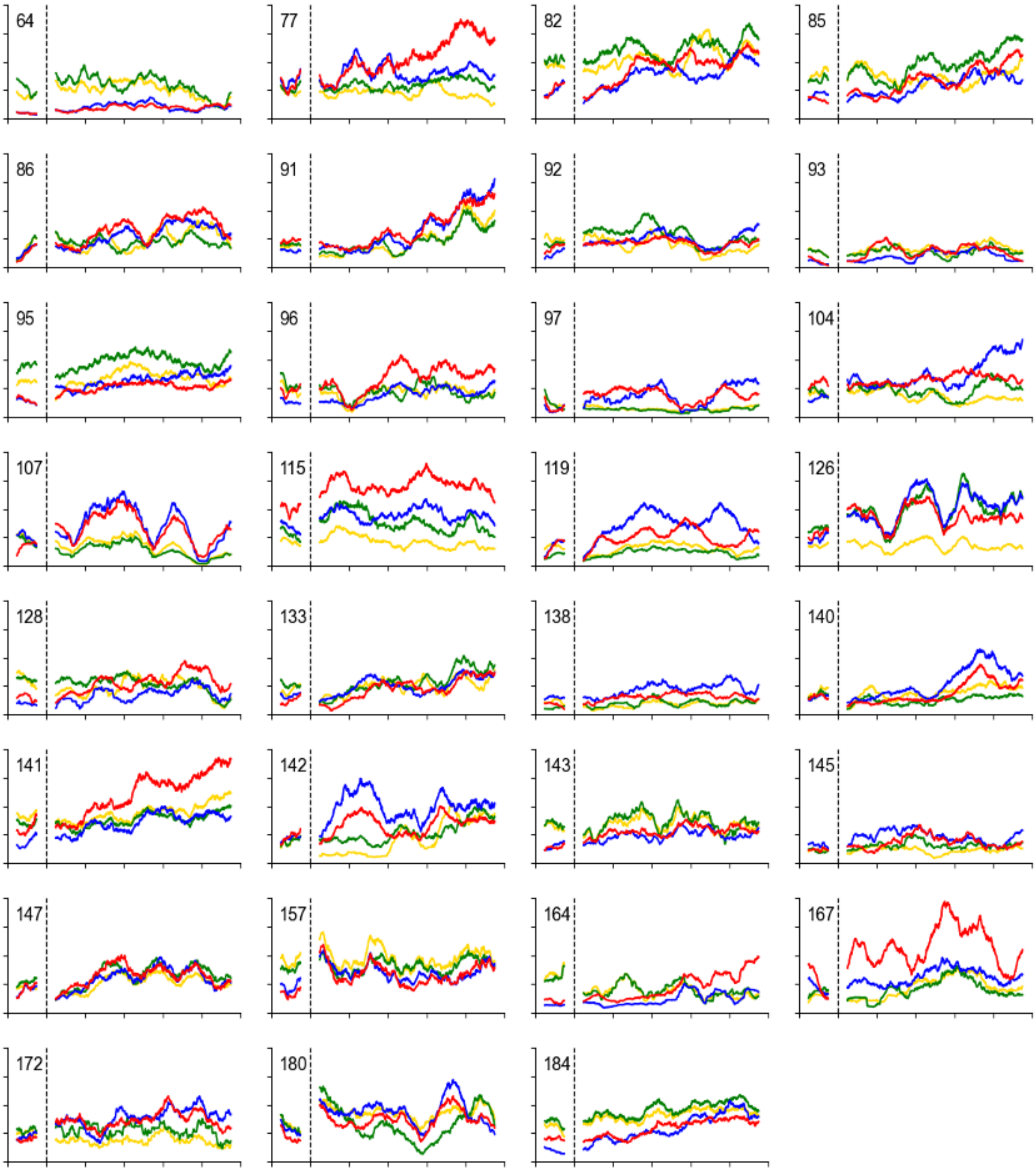
64
77
82
85
86
91
92
93
95
96
97
104
107
115
119
126
128
133
138
140
141
142
143
145
147
157
164
167
172
180
184

Cluster 3

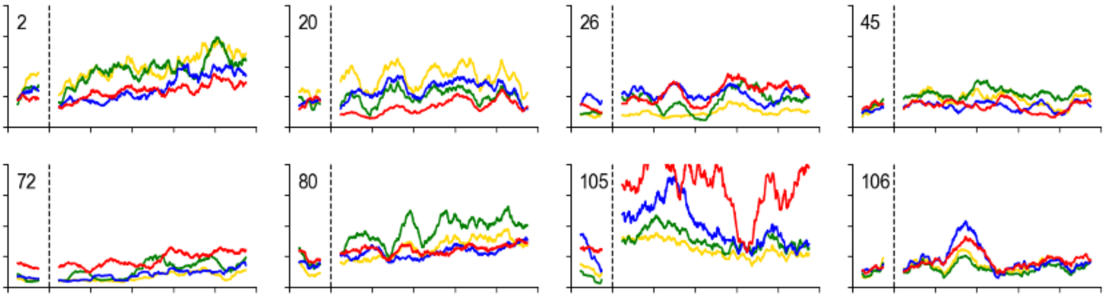
2
20
26
45
72
80
105
106

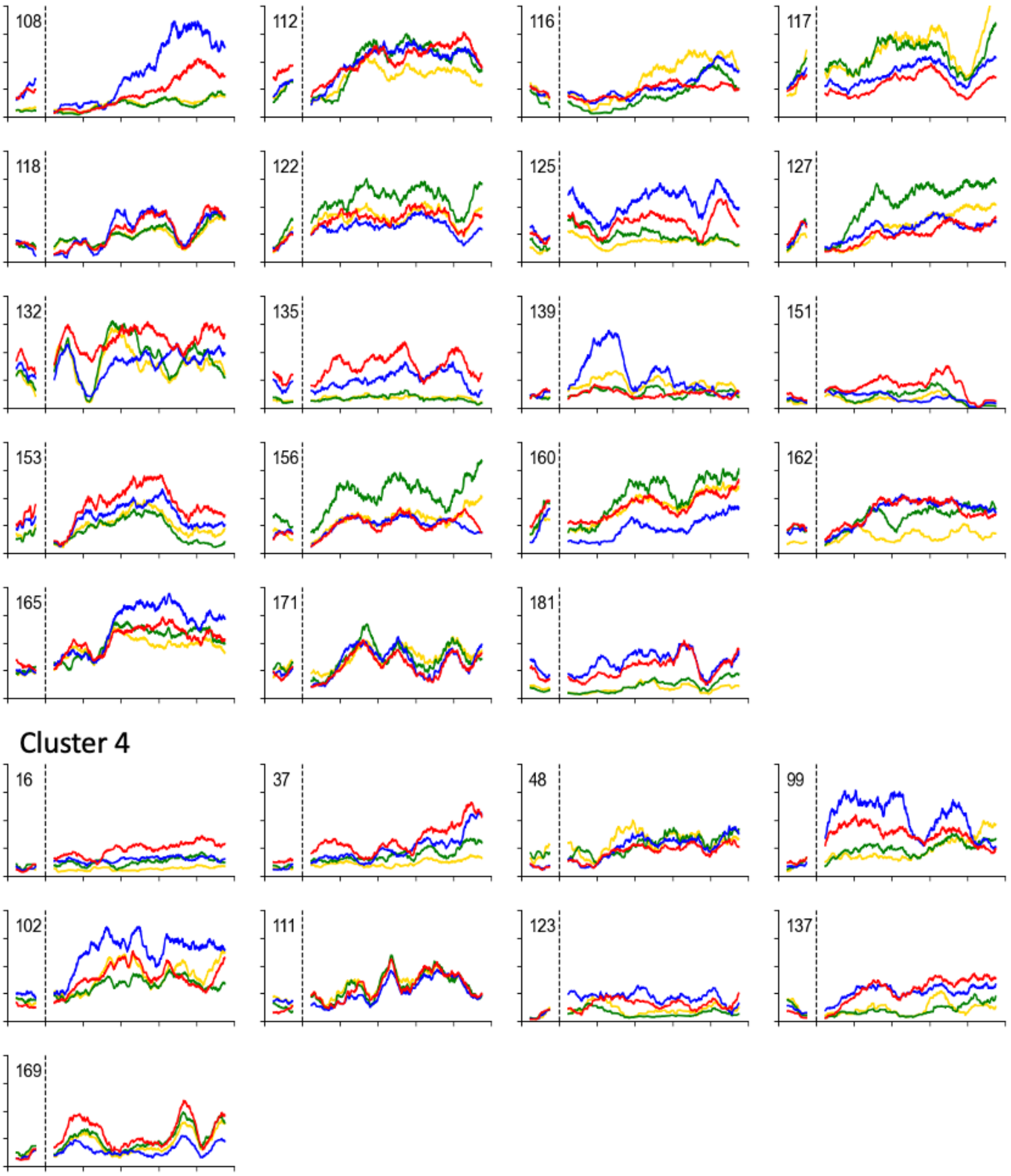


**Figure S6. Infant-wise time evolutions of displacement rate [m/min] for each limb**

ConA (red), UncA (blue), IpsiL (green), ContL (yellow). Panels are divided by the clusters of baseline ratio (main Figure 5, 6). The number written in each panel is infant's ID. 2-month-old infants are from 1 to 90, and 3-month-old infants are from 91 to 185. The maximum value of Y-axis is 20. Though displacement rates of some infants exceeded 20, this scale was most plausible to exhibit all time evolutions.

**Supplemenraty Table 1**

Tukey's HSD test across phases in 2-month-old ConA

| group 1 | group 2 | diff | 95% CI | p | Cohen's d |
|---|---|---|---|---|---|
| B | P1 | -0.735 | [-1.794, 0.325] | 0.178 | 0.358 |
| B | P2 | -0.108 | [-1.168, 0.951] | 0.999 | 0.049 |
| B | P3 | 0.292 | [-0.767, 1.351] | 0.938 | 0.134 |
| B | P4 | 0.732 | [-0.328, 1.791] | 0.182 | 0.325 |
| B | P5 | 1.087 | [0.028, 2.146] | **0.007** | 0.459 |
| P1 | P2 | 0.627 | [-0.433, 1.686] | 0.343 | 0.339 |
| P1 | P3 | 1.027 | [-0.032, 2.086] | 0.014 | 0.561 |
| P1 | P4 | 1.467 | [0.407, 2.526] | **<0.001** | 0.764 |
| P1 | P5 | 1.822 | [0.763, 2.881] | **<0.001** | 0.885 |
| P2 | P3 | 0.4 | [-0.659, 1.460] | 0.797 | 0.202 |
| P2 | P4 | 0.84 | [-0.219, 1.899] | 0.081 | 0.406 |
| P2 | P5 | 1.195 | [0.136, 2.255] | **0.002** | 0.544 |
| P3 | P4 | 0.44 | [-0.620, 1.499] | 0.725 | 0.214 |
| P3 | P5 | 0.795 | [-0.264, 1.854] | 0.115 | 0.364 |
| P4 | P5 | 0.355 | [-0.704, 1.415] | 0.867 | 0.157 |

**Supplemenraty Table 2**

Tukey's HSD test across phases in 3-month-old ConA

| group 1 | group 2 | diff | 95% CI | p | Cohen's d |
|---|---|---|---|---|---|
| B | P1 | -0.111 | [-1.564, 1.343] | 1 | 0.415 |
| B | P2 | 1.07 | [-0.383, 2.524] | 0.129 | 0.391 |
| B | P3 | 1.425 | [-0.029, 2.878] | 0.013 | 0.504 |
| B | P4 | 1.654 | [0.201, 3.108] | **0.002** | 0.631 |
| B | P5 | 1.8 | [0.347, 3.254] | **<0.001** | 0.648 |
| P1 | P2 | 1.181 | [-0.273, 2.635] | 0.068 | 0.39 |
| P1 | P3 | 1.535 | [0.082, 2.989] | **0.005** | 0.493 |
| P1 | P4 | 1.765 | [0.311, 3.219] | **<0.001** | 0.603 |
| P1 | P5 | 1.911 | [0.457, 3.365] | **<0.001** | 0.623 |
| P2 | P3 | 0.355 | [-1.099, 1.808] | 0.963 | 0.112 |
| P2 | P4 | 0.584 | [-0.870, 2.038] | 0.752 | 0.195 |
| P2 | P5 | 0.73 | [-0.724, 2.184] | 0.534 | 0.234 |
| P3 | P4 | 0.23 | [-1.224, 1.683] | 0.995 | 0.075 |
| P3 | P5 | 0.376 | [-1.078, 1.829] | 0.953 | 0.117 |
| P4 | P5 | 0.146 | [-1.308, 1.570] | 0.999 | 0.048 |

**Supplemenraty Table 3**

Tukey's HSD test across phases in 2-month-old UncA

| group 1 | group 2 | diff | 95% CI | p | Cohen's d |
|---|---|---|---|---|---|
| B | P1 | -0.823 | [-1.995 0.348] | 0.166 | 0.36 |
| B | P2 | -0.359 | [-1.530, 0.813] | 0.906 | 0.155 |

| | | | | | |
|---|---|---|---|---|---|
| B | P3 | 0.265 | [-0.906, 1.437] | 0.973 | 0.109 |
| B | P4 | 0.769 | [-0.403, 1.940] | 0.231 | 0.305 |
| B | P5 | 1.105 | [-0.067, 2.276] | 0.019 | 0.427 |
| P1 | P2 | 0.465 | [-0.707, 1.636] | 0.762 | 0.235 |
| P1 | P3 | 1.089 | [-0.083, 2.261] | 0.022 | 0.514 |
| P1 | P4 | 1.592 | [0.420, 2.764] | **<0.001** | 0.718 |
| P1 | P5 | 1.928 | [0.756, 3.100] | **<0.001** | 0.842 |
| P2 | P3 | 0.624 | [-0.548, 1.796] | 0.466 | 0.291 |
| P2 | P4 | 1.127 | [-0.045, 2.299] | 0.015 | 0.502 |
| P2 | P5 | 1.463 | [0.292, 2.635] | **<0.001** | 0.632 |
| P3 | P4 | 0.503 | [-0.669, 1.675] | 0.695 | 0.212 |
| P3 | P5 | 0.839 | [-0.332, 2.011] | 0.15 | 0.345 |
| P4 | P5 | 0.336 | [-0.835, 1.508] | 0.927 | 0.133 |

**Supplemenraty Table 4**

Tukey's HSD test across phases in 3-month-old UncA

| group 1 | group 2 | diff | 95% CI | p | Cohen's d |
|---|---|---|---|---|---|
| B | P1 | 0.04 | [-1.376, 1.455] | 1 | 0.015 |
| B | P2 | 1.178 | [-0.238, 2.593] | 0.057 | 0.44 |
| B | P3 | 1.412 | [-0.004, 2.827] | 0.0103 | 0.542 |
| B | P4 | 1.954 | [0.539, 3.369] | **<0.001** | 0.742 |
| B | P5 | 1.929 | [0.514, 3.344] | **<0.001** | 0.713 |
| P1 | P2 | 1.138 | [-0.278, 2.553] | 0.073 | 0.383 |
| P1 | P3 | 1.372 | [-0.044, 2.787] | 0.014 | 0.471 |
| P1 | P4 | 1.914 | [0.499, 3.330] | **<0.001** | 0.652 |
| P1 | P5 | 1.889 | [0.474, 3.305] | **<0.001** | 0.629 |
| P2 | P3 | 0.234 | [-1.182, 1.649] | 0.994 | 0.078 |
| P2 | P4 | 0.776 | [-0.639, 2.192] | 0.432 | 0.256 |
| P2 | P5 | 0.751 | [-0.664, 2.167] | 0.47 | 0.243 |
| P3 | P4 | 0.542 | [-0.873, 1.958] | 0.788 | 0.183 |
| P3 | P5 | 0.517 | [-0.898, 1.933] | 0.819 | 0.171 |
| P4 | P5 | -0.025 | [-1.440, 1.391] | 1 | 0.008 |

**Supplemenraty Table 5**

Tukey's HSD test across phases in 2-month-old IpsiL

| group 1 | group 2 | diff | 95% CI | p | Cohen's d |
|---|---|---|---|---|---|
| B | P1 | -0.848 | [-2.043, 0.347] | 0.158 | 0.374 |
| B | P2 | -0.7 | [-1.895, 0.495] | 0.354 | 0.297 |
| B | P3 | -0.373 | [-1.568, 0.822] | 0.898 | 0.16 |
| B | P4 | -0.061 | [-1.256, 1.134] | 1 | 0.025 |
| B | P5 | 0.062 | [-1.133, 1.257] | 1 | 0.025 |
| P1 | P2 | 0.148 | [-1.048, 1.342] | 0.998 | 0.067 |
| P1 | P3 | 0.474 | [-0.721, 1.669] | 0.761 | 0.219 |
| P1 | P4 | 0.787 | [-0.408, 1.982] | 0.227 | 0.343 |

| | | | | | |
|---|---|---|---|---|---|
| P1 | P5 | 0.91 | [-0.285, 2.105] | 0.105 | 0.381 |
| P2 | P3 | 0.327 | [-0.868, 1.522] | 0.94 | 0.144 |
| P2 | P4 | 0.639 | [-0.555, 1.834] | 0.46 | 0.268 |
| P2 | P5 | 0.763 | [-0.432, 1.958] | 0.259 | 0.308 |
| P3 | P4 | 0.312 | [-0.883, 1.507] | 0.95 | 0.132 |
| P3 | P5 | 0.436 | [-0.759, 1.631] | 0.82 | 0.177 |
| P4 | P5 | 0.123 | [-1.072, 1.318] | 0.999 | 0.048 |

**Supplemenraty Table 6**

Tukey's HSD test across phases in 3-month-old IpsiL

| group 1 | group 2 | diff | 95% CI | p | Cohen's d |
|---|---|---|---|---|---|
| B | P1 | -0.427 | [-1.904, 1.05] | 0.925 | 0.175 |
| B | P2 | 0.459 | [1.018, 1.936] | 0.901 | 0.177 |
| B | P3 | 0.729 | [-0.749, 2.206] | 0.554 | 0.256 |
| B | P4 | 0.808 | [-0.669, 2.285] | 0.435 | 0.294 |
| B | P5 | 1.336 | [-0.141, 2.813] | 0.028 | 0.425 |
| P1 | P2 | 0.886 | [-0.591, 2.363] | 0.328 | 0.331 |
| P1 | P3 | 1.155 | [-0.322, 2.633] | 0.088 | 0.395 |
| P1 | P4 | 1.235 | [-0.242, 2.712] | 0.055 | 0.436 |
| P1 | P5 | 1.763 | [0.286, 3.240] | **<0.001** | 0.549 |
| P2 | P3 | 0.27 | [-1.207, 1.747] | 0.99 | 0.088 |
| P2 | P4 | 0.349 | [-1.128, 1.826] | 0.968 | 0.118 |
| P2 | P5 | 0.877 | [-0.600, 2.355] | 0.339 | 0.263 |
| P3 | P4 | 0.079 | [-1.398, 1.556] | 1 | 0.025 |
| P3 | P5 | 0.608 | [-0.869, 2.085] | 0.732 | 0.172 |
| P4 | P5 | 0.529 | [-0.948, 2.006] | 0.832 | 0.153 |

**Supplemenraty Table 7**

Tukey's HSD test across phases in 2-month-old ContL

| group 1 | group 2 | diff | 95% CI | p | Cohen's d |
|---|---|---|---|---|---|
| B | P1 | -0.841 | [-1.920, 0.238] | 0.091 | 0.417 |
| B | P2 | -0.604 | [-1.682, 0.475] | 0.408 | 0.276 |
| B | P3 | -0.642 | [-1.720, 0.437] | 0.338 | 0.308 |
| B | P4 | -0.39 | [-1.469, 0.689] | 0.826 | 0.179 |
| B | P5 | -0.178 | [-1.257, 0.901] | 0.994 | 0.077 |
| P1 | P2 | 0.237 | [-0.841, 1.316] | 0.976 | 0.118 |
| P1 | P3 | 0.2 | [-0.879, 1.278] | 0.989 | 0.105 |
| P1 | P4 | 0.451 | [-0628, 1.530] | 0.719 | 0.224 |
| P1 | P5 | 0.663 | [-0.416, 1.742] | 0.301 | 0.308 |
| P2 | P3 | -0.038 | [-1.117, 1.041] | 1 | 0.018 |
| P2 | P4 | 0.213 | [-0.865, 1.292] | 0.985 | 0.098 |
| P2 | P5 | 0.425 | [-0.654, 1.504] | 0.766 | 0.184 |
| P3 | P4 | 0.251 | [-0.828, 1.330] | 0.97 | 0.121 |
| P3 | P5 | 0.463 | [-0.616, 1.542] | 0.695 | 0.209 |

| | | | | | |
|---|---|---|---|---|---|
| P4 | P5 | 0.212 | [-0.867, 1.291] | 0.986 | 0.092 |

**Supplemenraty Table 8**

Tukey's HSD test across phases in 3-month-old ContL

| group 1 | group 2 | diff | 95% CI | p | Cohen's d |
|---|---|---|---|---|---|
| B | P1 | -0.422 | [-1.709, 0.865] | 0.878 | 0.185 |
| B | P2 | 0.123 | [-1.164, 1.41] | 1 | 0.053 |
| B | P3 | 0.478 | [-0.809, 1.765] | 0.809 | 0.193 |
| B | P4 | 0.635 | [-0.652, 1.922] | 0.554 | 0.266 |
| B | P5 | 1.051 | [-0.236, 2.338] | 0.066 | 0.363 |
| P1 | P2 | 0.545 | [-0.742, 1.832] | 0.708 | 0.233 |
| P1 | P3 | 0.9 | [-0.387, 2.187] | 0.171 | 0.358 |
| P1 | P4 | 1.057 | [-0.230, 2.344] | 0.063 | 0.436 |
| P1 | P5 | 1.473 | [0.186, 2.760] | **0.002** | 0.503 |
| P2 | P3 | 0.355 | [-0.932, 1.642] | 0.938 | 0.14 |
| P2 | P4 | 0.512 | [-0.775, 1.799] | 0.76 | 0.21 |
| P2 | P5 | 0.928 | [-0.359, 2.215] | 0.145 | 0.315 |
| P3 | P4 | 0.157 | [-1.130, 1.444] | 0.999 | 0.06 |
| P3 | P5 | 0.573 | [-0.714, 1.860] | 0.661 | 0.186 |
| P4 | P5 | 0.416 | [-0.871, 1.703] | 0.884 | 0.138 |

**Supplementary Table 9**

Tukey's HSD test across phases in Cluster 1 ConA (not conducted)

| group 1 | group 2 | diff | 95% CI | p | Cohen's d |
|---|---|---|---|---|---|
| B | P1 | | | | |
| B | P2 | | | | |
| B | P3 | | | | |
| B | P4 | | | | |
| B | P5 | | | | |
| P1 | P2 | | | | |
| P1 | P3 | | | | |
| P1 | P4 | | | | |
| P1 | P5 | | | | |
| P2 | P3 | | | | |
| P2 | P4 | | | | |
| P2 | P5 | | | | |
| P3 | P4 | | | | |
| P3 | P5 | | | | |
| P4 | P5 | | | | |

**Supplementary Table 10**

Tukey's HSD test across phases in Cluster 2 ConA

| group 1 | group 2 | diff | 95% CI | p | Cohen's d |
|---|---|---|---|---|---|
| B | P1 | 0.794 | [-1.165, 2.753] | 0.741 | 0.358 |
| B | P2 | 1.728 | [-0.231, 3.687] | 0.035 | 0.755 |
| B | P3 | 2.187 | [0.228, 4.146] | **0.003** | 0.871 |
| B | P4 | 3.019 | [1.060, 4.979] | **<0.001** | 1.151 |
| B | P5 | 3.135 | [1.176, 5.094] | **<0.001** | 1.17 |
| P1 | P2 | 0.934 | [-1.025, 2.893] | 0.586 | 0.364 |
| P1 | P3 | 1.394 | [-0.566, 3.352] | 0.154 | 0.503 |
| P1 | P4 | 2.226 | [0.267, 4.185] | **0.002** | 0.775 |
| P1 | P5 | 2.341 | [0.382, 4.300] | **0.001** | 0.801 |
| P2 | P3 | 0.459 | [-1.500, 2.419] | 0.968 | 0.162 |
| P2 | P4 | 1.292 | [-0.667, 3.251] | 0.223 | 0.441 |
| P2 | P5 | 1.407 | [-0.552, 3.366] | 0.146 | 0.472 |
| P3 | P4 | 0.832 | [-1.127, 2.791] | 0.7 | 0.268 |
| P3 | P5 | 0.948 | [-1.012, 2.907] | 0.57 | 0.301 |
| P4 | P5 | 0.115 | [-1.844, 2.074] | 1 | 0.036 |

**Supplementary Table 11**

Tukey's HSD test across phases in Cluster 3 ConA

| group 1 | group 2 | diff | 95% CI | p | Cohen's d |
|---|---|---|---|---|---|
| B | P1 | -0.021 | [-2.927, 2.886] | 1 | 0.008 |
| B | P2 | 1.816 | [-1.091, 4.723] | 0.272 | 0.646 |

| | | | | | |
|---|---|---|---|---|---|
| B | P3 | 2.737 | [-0.170, 5.644] | 0.019 | 0.973 |
| B | P4 | 2.267 | [-0.640, 5.174] | 0.087 | 1.012 |
| B | P5 | 2.743 | [-0.164, 5.649] | 0.018 | 1.037 |
| P1 | P2 | 1.837 | [-1.070, 4.743] | 0.26 | 0.533 |
| P1 | P3 | 2.758 | [-0.149, 5.664] | 0.017 | 0.799 |
| P1 | P4 | 2.288 | [-0.619, 5.194] | 0.082 | 0.762 |
| P1 | P5 | 2,763 | [-0.144, 5.670] | 0.017 | 0.834 |
| P2 | P3 | 0.921 | [-1.986, 3.828] | 0.887 | 0.254 |
| P2 | P4 | 0.451 | [-2.456, 3.358] | 0.995 | 0.141 |
| P2 | P5 | 0.927 | [-1.980, 3.833] | 0.884 | 0.265 |
| P3 | P4 | -0.47 | [-3.377, 2.437] | 0.994 | 0.147 |
| P3 | P5 | 0.006 | [-2.901, 2.912] | 1 | 0.002 |
| P4 | P5 | 0.476 | [-2.431, 3.382] | 0.993 | 0.156 |

**Supplementary Table 12**

Tukey's HSD test across phases in Cluster 4 ConA

| group 1 | group 2 | diff | 95% CI | p | Cohen's d |
|---|---|---|---|---|---|
| B | P1 | 2.066 | [-0.959, 5.091] | 0.163 | 1.462 |
| B | P2 | 3.494 | [0.469, 6.519] | **0.002** | 2.366 |
| B | P3 | 3.616 | [0.591, 6.641] | **0.001** | 2.618 |
| B | P4 | 4.839 | [1.814, 7.865] | **<0.001** | 3.214 |
| B | P5 | 4.536 | [1.511, 7.561] | **<0.001** | 3.073 |
| P1 | P2 | 1.428 | [-1.598, 4.453] | 0.548 | 0.737 |
| P1 | P3 | 1.55 | [-1.476, 4.575] | 0.457 | 0.831 |
| P1 | P4 | 2.773 | [-0.252, 5.798] | 0.023 | 1.415 |
| P1 | P5 | 2.47 | [-0.555, 5.495] | 0.057 | 1.275 |
| P2 | P3 | 0.122 | [-2.903, 3.147] | 1 | 0.064 |
| P2 | P4 | 1.346 | [-1.680, 4.371] | 0.61 | 0.671 |
| P2 | P5 | 1.042 | [-1.983, 4.068] | 0.82 | 0.526 |
| P3 | P4 | 1.223 | [-1.802, 4.249] | 0.7 | 0.632 |
| P3 | P5 | 0.92 | [-2.105, 3.945] | 0.884 | 0.481 |
| P4 | P5 | -0.303 | [-3.329, 2.722] | 0.999 | 0.151 |

**Supplementary Table 13**

Tukey's HSD test across clusters in Baseline ConA

| group 1 | group 2 | diff | 95% CI | p | Cohen's d |
|---|---|---|---|---|---|
| C1 | C2 | -2.15 | [-3.313, -0.987] | **<0.001** | 0.973 |
| C1 | C3 | -0.96 | [-2.387, 0.468] | 0.15 | 0.433 |
| C1 | C4 | -3.512 | [-5.806, -1.218] | **<0.001** | 1.553 |
| C2 | C3 | 1.19 | [-0.403, 2.783] | 0.089 | 0.662 |
| C2 | C4 | -1.363 | [-3.763, 1.038] | 0.281 | 0.776 |
| C3 | C4 | -2.553 | [-5.092, -0.014] | **0.0095** | 1.739 |

**Supplementary Table 14**

Tukey's HSD test across clusters in Play 1 ConA (not conducted)

| group 1 | group 2 | diff | 95% CI | p | Cohen's d |
|---|---|---|---|---|---|
| C1 | C2 | | | | |
| C1 | C3 | | | | |
| C1 | C4 | | | | |
| C2 | C3 | | | | |
| C2 | C4 | | | | |
| C3 | C4 | | | | |

**Supplementary Table 15**

Tukey's HSD test across clusters in Play 2 ConA (not conducted)

| group 1 | group 2 | diff | 95% CI | p | Cohen's d |
|---|---|---|---|---|---|
| C1 | C2 | | | | |
| C1 | C3 | | | | |
| C1 | C4 | | | | |
| C2 | C3 | | | | |
| C2 | C4 | | | | |
| C3 | C4 | | | | |

**Supplementary Table 16**

Tukey's HSD test across clusters in Play 3 ConA

| group 1 | group 2 | diff | 95% CI | p | Cohen's d |
|---|---|---|---|---|---|
| C1 | C2 | 0.504 | [-0.976, 1.984] | 0.705 | 0.202 |
| C1 | C3 | 2.244 | [0.427, 4.061] | **<0.001** | 0.875 |
| C1 | C4 | 0.57 | [-2.349, 3.490] | 0.927 | 0.261 |
| C2 | C3 | 1.74 | [-0.287, 3.768] | 0.037 | 0.536 |
| C2 | C4 | 0.066 | [-2.988, 3.121] | 1 | 0.023 |
| C3 | C4 | -1.674 | [-4.905, 1.558] | 0.361 | 0.508 |

**Supplementary Table 17**

Tukey's HSD test across clusters in Play 4 ConA

| group 1 | group 2 | diff | 95% CI | p | Cohen's d |
|---|---|---|---|---|---|
| C1 | C2 | 1.102 | [-0.294, 2.497] | 0.064 | 0.442 |
| C1 | C3 | 1.539 | [-0.174, 3.252] | 0.026 | 0.689 |
| C1 | C4 | 1.559 | [-1.193, 4.311] | 0.282 | 0.746 |
| C2 | C3 | 0.438 | [-1.474, 2.349] | 0.888 | 0.144 |
| C2 | C4 | 0.457 | [-2.423, 3.337] | 0.959 | 0.15 |
| C3 | C4 | 0.02 | [-3.027, 3.066] | 1 | 0.008 |

**Supplementary Table 18**

Tukey's HSD test across clusters in Play 5 ConA (not conducted)

| group 1 | group 2 | diff | 95% CI | p | Cohen's d |
|---|---|---|---|---|---|
| C1 | C2 | | | | |
| C1 | C3 | | | | |

| | |
|---|---|
| C1 | C4 |
| C2 | C3 |
| C2 | C4 |
| C3 | C4 |

**Supplementary Table 19**

Tukey's HSD test across phases in Cluster 1 UncA (not conducted)

| group 1 | group 2 | diff | 95% CI | p | Cohen's d |
|---|---|---|---|---|---|
| B | P1 | | | | |
| B | P2 | | | | |
| B | P3 | | | | |
| B | P4 | | | | |
| B | P5 | | | | |
| P1 | P2 | | | | |
| P1 | P3 | | | | |
| P1 | P4 | | | | |
| P1 | P5 | | | | |
| P2 | P3 | | | | |
| P2 | P4 | | | | |
| P2 | P5 | | | | |
| P3 | P4 | | | | |
| P3 | P5 | | | | |
| P4 | P5 | | | | |

**Supplementary Table 20**

Tukey's HSD test across phases in Cluster 2 UncA

| group 1 | group 2 | diff | 95% CI | p | Cohen's d |
|---|---|---|---|---|---|
| B | P1 | 0.619 | [-1.026, 2.265] | 0.796 | 0.328 |
| B | P2 | 1.88 | [0.234, 3.525] | **0.002** | 0.869 |
| B | P3 | 2.194 | [0.549, 3.840] | **<0.001** | 1.18 |
| B | P4 | 3.037 | [1.391, 4.682] | **<0.001** | 1.409 |
| B | P5 | 3.317 | [1.672, 4.963] | **<0.001** | 1.463 |
| P1 | P2 | 1.26 | [-0.385, 2.906] | 0.1 | 0.525 |
| P1 | P3 | 1.575 | [-0.071, 3.220] | 0.016 | 0.74 |
| P1 | P4 | 2.418 | [0.772, 4.063] | **<0.001** | 1.01 |
| P1 | P5 | 2.698 | [1.052, 4.344] | **<0.001** | 1.082 |
| P2 | P3 | 0.315 | [-1.331, 1.960] | 0.987 | 0.132 |
| P2 | P4 | 1.157 | [-0.488, 2.803] | 0.163 | 0.442 |
| P2 | P5 | 1.438 | [-0.208, 3.083] | 0.038 | 0.531 |
| P3 | P4 | 0.843 | [-0.803, 2.488] | 0.506 | 0.355 |
| P3 | P5 | 1.123 | [-0.522, 2.769] | 0.19 | 0.454 |
| P4 | P5 | 0.28 | [-1.365, 1.926] | 0.992 | 0.104 |

**Supplementary Table 21**

Tukey's HSD test across phases in Cluster 3 UncA

| group 1 | group 2 | diff | 95% CI | p | Cohen's d |
|---|---|---|---|---|---|
| B | P1 | 0.182 | [-2.513, 2.877] | 1 | 0.089 |
| B | P2 | 1.917 | [-0.778, 4.612] | 0.15 | 0.855 |

| | | | | | |
|---|---|---|---|---|---|
| B | P3 | 2.905 | [0.210, 5.599] | **0.004** | 1.154 |
| B | P4 | 2.763 | [0.068, 5.458] | **0.008** | 1.081 |
| B | P5 | 2.68 | [-0.015, 5.375] | 0.011 | 1.035 |
| P1 | P2 | 1.735 | [-0.960, 4.430] | 0.241 | 0.64 |
| P1 | P3 | 2.723 | [0.028, 5.418] | **0.009** | 0.925 |
| P1 | P4 | 2.581 | [-0.114, 5.276] | 0.016 | 0.867 |
| P1 | P5 | 2.498 | [-0.197, 5.193] | 0.022 | 0.831 |
| P2 | P3 | 0.987 | [-1.707, 3.682] | 0.809 | 0.32 |
| P2 | P4 | 0.846 | [-1.849, 3.540] | 0.891 | 0.272 |
| P2 | P5 | 0.763 | [-1.932, 3.457] | 0.927 | 0.243 |
| P3 | P4 | -0.142 | [-2.837, 2.553] | 1 | 0.043 |
| P3 | P5 | -0.225 | [-2.920, 2.470] | 1 | 0.067 |
| P4 | P5 | -0.083 | [-2.778, 2.612] | 1 | 0.025 |

**Supplementary Table 22**

Tukey's HSD test across phases in Cluster 4 UncA

| group 1 | group 2 | diff | 95% CI | p | Cohen's d |
|---|---|---|---|---|---|
| B | P1 | 1.999 | [-3.509, 7.507] | 0.785 | 0.949 |
| B | P2 | 3.7 | [-1.808, 9.207] | 0.177 | 1.134 |
| B | P3 | 3.716 | [-1.792, 9.223] | 0.173 | 1.498 |
| B | P4 | 4.71 | [-0.798, 10.217] | 0.04 | 1.736 |
| B | P5 | 3.977 | [-1.531, 9.485] | 0.122 | 1.666 |
| P1 | P2 | 1.701 | [-3.807, 7.209] | 0.878 | 0.455 |
| P1 | P3 | 1.717 | [-3.791, 7.224] | 0.873 | 0.557 |
| P1 | P4 | 2.711 | [-2.797, 8.218] | 0.502 | 0.829 |
| P1 | P5 | 1.978 | [-3.530, 7.486] | 0.793 | 0.658 |
| P2 | P3 | 0.016 | [-5.492, 5.524] | 1 | 0.004 |
| P2 | P4 | 1.01 | [-4.498, 6.517] | 0.986 | 0.246 |
| P2 | P5 | 0.277 | [-5.231, 5.785] | 1 | 0.071 |
| P3 | P4 | 0.994 | [-4.514, 6.501] | 0.987 | 0.282 |
| P3 | P5 | 0.261 | [-5.247, 5.769] | 1 | 0.08 |
| P4 | P5 | -0.733 | [-6.240, 4.775] | 0.997 | 0.212 |

**Supplementary Table 23**

Tukey's HSD test across clusters in Baseline UncA

| group 1 | group 2 | diff | 95% CI | p | Cohen's d |
|---|---|---|---|---|---|
| C1 | C2 | -2.489 | [-3.658, -1.320] | <0.001 | 1.108 |
| C1 | C3 | -1.385 | [-2.821, 0.050] | 0.014 | 0.601 |
| C1 | C4 | -3.347 | [-5.653, -1.041] | **<0.001** | 1.386 |
| C2 | C3 | 1.104 | [-0.498, 2.705] | 0.134 | 0.734 |
| C2 | C4 | -0.858 | [-3.271, 1.555] | 0.676 | 0.568 |
| C3 | C4 | -1.962 | [-4.514, 0.591] | 0.076 | 1.51 |

**Supplementary Table 24**

Tukey's HSD test across clusters in Play 1 UncA (not conducted)

| group 1 | group 2 | diff | 95% CI | p | Cohen's d |
|---|---|---|---|---|---|
| C1 | C2 | | | | |
| C1 | C3 | | | | |
| C1 | C4 | | | | |
| C2 | C3 | | | | |
| C2 | C4 | | | | |
| C3 | C4 | | | | |

**Supplementary Table 25**

Tukey's HSD test across clusters in Play 2 UncA (not conducted)

| group 1 | group 2 | diff | 95% CI | p | Cohen's d |
|---|---|---|---|---|---|
| C1 | C2 | | | | |
| C1 | C3 | | | | |
| C1 | C4 | | | | |
| C2 | C3 | | | | |
| C2 | C4 | | | | |
| C3 | C4 | | | | |

**Supplementary Table 26**

Tukey's HSD test across clusters in Play 3 UncA

| group 1 | group 2 | diff | 95% CI | p | Cohen's d |
|---|---|---|---|---|---|
| C1 | C2 | 0.264 | [-1.178, 1.706] | 0.939 | 0.11 |
| C1 | C3 | 2.078 | [0.308, 3.849] | **0.002** | 0.772 |
| C1 | C4 | 0.928 | [-1.917, 3.772] | 0.732 | 0.358 |
| C2 | C3 | 1.815 | [-0.161, 3.790] | 0.022 | 0.699 |
| C2 | C4 | 0.664 | [-2.312, 3.640] | 0.895 | 0.285 |
| C3 | C4 | -1.151 | [-4.299, 1.998] | 0.657 | 0.349 |

**Supplementary Table 27**

Tukey's HSD test across clusters in Play 4 UncA (not conducted)

| group 1 | group 2 | diff | 95% CI | p | Cohen's d |
|---|---|---|---|---|---|
| C1 | C2 | | | | |
| C1 | C3 | | | | |
| C1 | C4 | | | | |
| C2 | C3 | | | | |
| C2 | C4 | | | | |
| C3 | C4 | | | | |

**Supplementary Table 28**

Tukey's HSD test across clusters in Play 5 UncA (not conducted)

| group 1 | group 2 | diff | 95% CI | p | Cohen's d |
|---|---|---|---|---|---|
| C1 | C2 | | | | |
| C1 | C3 | | | | |

| | |
|---|---|
| C1 | C4 |
| C2 | C3 |
| C2 | C4 |
| C3 | C4 |

**Supplementary Table 29**

Tukey's HSD test across phases in Cluster 1 IpsiL (not conducted)

| group 1 | group 2 | diff | 95% CI | p | Cohen's d |
|---|---|---|---|---|---|
| B | P1 | | | | |
| B | P2 | | | | |
| B | P3 | | | | |
| B | P4 | | | | |
| B | P5 | | | | |
| P1 | P2 | | | | |
| P1 | P3 | | | | |
| P1 | P4 | | | | |
| P1 | P5 | | | | |
| P2 | P3 | | | | |
| P2 | P4 | | | | |
| P2 | P5 | | | | |
| P3 | P4 | | | | |
| P3 | P5 | | | | |
| P4 | P5 | | | | |

**Supplementary Table 30**

Tukey's HSD test across phases in Cluster 2 IpsiL (not conducted)

| group 1 | group 2 | diff | 95% CI | p | Cohen's d |
|---|---|---|---|---|---|
| B | P1 | | | | |
| B | P2 | | | | |
| B | P3 | | | | |
| B | P4 | | | | |
| B | P5 | | | | |
| P1 | P2 | | | | |
| P1 | P3 | | | | |
| P1 | P4 | | | | |
| P1 | P5 | | | | |
| P2 | P3 | | | | |
| P2 | P4 | | | | |
| P2 | P5 | | | | |
| P3 | P4 | | | | |
| P3 | P5 | | | | |
| P4 | P5 | | | | |

**Supplementary Table 31**

Tukey's HSD test across phases in Cluster 3 IpsiL

| group 1 | group 2 | diff | 95% CI | p | Cohen's d |
|---|---|---|---|---|---|
| B | P1 | 0.647 | [-2.531, 3.825] | 0.982 | 0.303 |
| B | P2 | 2.572 | [-0.606, 5.750] | 0.067 | 0.88 |

| | | | | | |
|---|---|---|---|---|---|
| B | P3 | 3.487 | [0.309, 6.665] | **0.003** | 1.156 |
| B | P4 | 3.076 | [-0.102, 6.254] | 0.014 | 1.075 |
| B | P5 | 3.409 | [0.231, 6.587] | **0.004** | 1.056 |
| P1 | P2 | 1.924 | [-1.254, 5.102] | 0.306 | 0.605 |
| P1 | P3 | 2.839 | [-0.339, 6.017] | 0.031 | 0.869 |
| P1 | P4 | 2.428 | [-0.750, 5.606] | 0.099 | 0.777 |
| P1 | P5 | 2.762 | [-0.416, 5.940] | 0.039 | 0.797 |
| P2 | P3 | 0.915 | [-2.263, 4.093] | 0.922 | 0.239 |
| P2 | P4 | 0.504 | [-2.674, 3.682] | 0.994 | 0.136 |
| P2 | P5 | 0.838 | [-2.340, 4.016] | 0.945 | 0.21 |
| P3 | P4 | -0.411 | [-3.589, 2.767] | 0.998 | 0.109 |
| P3 | P5 | -0.077 | [-3.255, 3.101] | 1 | 0.019 |
| P4 | P5 | 0.334 | [-2.845, 3.511] | 0.999 | 0.084 |

**Supplementary Table 32**

Tukey's HSD test across phases in Cluster 4 IpsiL

| group 1 | group 2 | diff | 95% CI | p | Cohen's d |
|---|---|---|---|---|---|
| B | P1 | 0.446 | [-2.811, 3.702] | 0.996 | 0.445 |
| B | P2 | 1.08 | [-2.176, 4.336] | 0.842 | 0.753 |
| B | P3 | 1.432 | [-1.825, 4.688] | 0.621 | 0.773 |
| B | P4 | 2.778 | [-0.479, 6.034] | 0.041 | 1.349 |
| B | P5 | 2.675 | [-0.582, 5.931] | 0.054 | 1.757 |
| P1 | P2 | 0.634 | [-2.622, 3.890] | 0.982 | 0.433 |
| P1 | P3 | 0.986 | [-2.270, 4.242] | 0.886 | 0.526 |
| P1 | P4 | 2.332 | [-0.924, 5.588] | 0.128 | 1.121 |
| P1 | P5 | 2.229 | [-1.027, 5.485] | 0.162 | 1.438 |
| P2 | P3 | 0.352 | [-2.904, 3.608] | 0.999 | 0.165 |
| P2 | P4 | 1.698 | [-1.558, 4.954] | 0.437 | 0.732 |
| P2 | P5 | 1.595 | [-1.662, 4.851] | 0.507 | 0.858 |
| P3 | P4 | 1.346 | [-1.910, 4.602] | 0.68 | 0.518 |
| P3 | P5 | 1.243 | [-2.013, 4.499] | 0.748 | 0.565 |
| P4 | P5 | -0.103 | [-3.359, 3.153] | 1 | 0.043 |

**Supplementary Table 33**

Tukey's HSD test across clusters in Baseline IpsiL

| group 1 | group 2 | diff | 95% CI | p | Cohen's d |
|---|---|---|---|---|---|
| C1 | C2 | -0.798 | [-2.048, 0.452] | 0.186 | 0.336 |
| C1 | C3 | -1.957 | [-3.492, -0.423] | **<0.001** | 0.838 |
| C1 | C4 | -3.075 | [-5.541, -0.610] | **<0.001** | 1.287 |
| C2 | C3 | -1.159 | [-2.871, 0.553] | 0.145 | 0.577 |
| C2 | C4 | -2.277 | [-4.857, 0.303] | 0.03 | 1.128 |
| C3 | C4 | -1.118 | [-3.847, 1.611] | 0.568 | 0.706 |

**Supplementary Table 34**

Tukey's HSD test across clusters in Play 1 IpsiL

| group 1 | group 2 | diff | 95% CI | p | Cohen's d |
|---|---|---|---|---|---|
| C1 | C2 | 0.29 | [-0.997, 1.578] | 0.892 | 0.124 |
| C1 | C3 | -0.038 | [-1.619, 1.543] | 1 | 0.017 |
| C1 | C4 | -1.358 | [-3.897, 1.182] | 0.333 | 0.628 |
| C2 | C3 | -0.328 | [-2.092, 1.435] | 0.938 | 0.13 |
| C2 | C4 | -1.648 | [-4.305, 1.009] | 0.208 | 0.69 |
| C3 | C4 | -1.32 | [-4.131, 1.491] | 0.45 | 0.593 |

**Supplementary Table 35**

Tukey's HSD test across clusters in Play 2 IpsiL

| group 1 | group 2 | diff | 95% CI | p | Cohen's d |
|---|---|---|---|---|---|
| C1 | C2 | 0.65 | [-0.754, 2.054] | 0.463 | 0.286 |
| C1 | C3 | 1.733 | [0.009, 3.456] | **0.0095** | 0.676 |
| C1 | C4 | -0.877 | [-3.646, 1.892] | 0.749 | 0.413 |
| C2 | C3 | 1.083 | [-0.840, 3.006] | 0.287 | 0.358 |
| C2 | C4 | -1.527 | [-4.424, 1.371] | 0.346 | 0.63 |
| C3 | C4 | -2.61 | [-5.675, 0.455] | 0.039 | 0.769 |

**Supplementary Table 36**

Tukey's HSD test across clusters in Play 3 IpsiL

| group 1 | group 2 | diff | 95% CI | p | Cohen's d |
|---|---|---|---|---|---|
| C1 | C2 | 0.553 | [-0.954, 2.061] | 0.654 | 0.226 |
| C1 | C3 | 2.435 | [0.584, 4.286] | **<0.001** | 0.888 |
| C1 | C4 | -0.738 | [-3.712, 2.235] | 0.862 | 0.313 |
| C2 | C3 | 1.882 | [-0.184, 3.947] | 0.023 | 0.595 |
| C2 | C4 | -1.292 | [-4.403, 1.820] | 0.558 | 0.491 |
| C3 | C4 | -3.173 | [-6.465, 0.119] | 0.014 | 0.879 |

**Supplementary Table 37**

Tukey's HSD test across clusters in Play 4 IpsiL (not conducted)

| group 1 | group 2 | diff | 95% CI | p | Cohen's d |
|---|---|---|---|---|---|
| C1 | C2 | | | | |
| C1 | C3 | | | | |
| C1 | C4 | | | | |
| C2 | C3 | | | | |
| C2 | C4 | | | | |
| C3 | C4 | | | | |

**Supplementary Table 38**

Tukey's HSD test across clusters in Play 5 IpsiL (not conducted)

| group 1 | group 2 | diff | 95% CI | p | Cohen's d |
|---|---|---|---|---|---|
| C1 | C2 | | | | |
| C1 | C3 | | | | |

| | |
|---|---|
| C1 | C4 |
| C2 | C3 |
| C2 | C4 |
| C3 | C4 |

**Supplementary Table 39**

Tukey's HSD test across phases in Cluster 1 ContL (not conducted)

| group 1 | group 2 | diff | 95% CI | p | Cohen's d |
|---|---|---|---|---|---|
| B | P1 | | | | |
| B | P2 | | | | |
| B | P3 | | | | |
| B | P4 | | | | |
| B | P5 | | | | |
| P1 | P2 | | | | |
| P1 | P3 | | | | |
| P1 | P4 | | | | |
| P1 | P5 | | | | |
| P2 | P3 | | | | |
| P2 | P4 | | | | |
| P2 | P5 | | | | |
| P3 | P4 | | | | |
| P3 | P5 | | | | |
| P4 | P5 | | | | |

**Supplementary Table 40**

Tukey's HSD test across phases in Cluster 2 ContL (not conducted)

| group 1 | group 2 | diff | 95% CI | p | Cohen's d |
|---|---|---|---|---|---|
| B | P1 | | | | |
| B | P2 | | | | |
| B | P3 | | | | |
| B | P4 | | | | |
| B | P5 | | | | |
| P1 | P2 | | | | |
| P1 | P3 | | | | |
| P1 | P4 | | | | |
| P1 | P5 | | | | |
| P2 | P3 | | | | |
| P2 | P4 | | | | |
| P2 | P5 | | | | |
| P3 | P4 | | | | |
| P3 | P5 | | | | |
| P4 | P5 | | | | |

**Supplementary Table 41**

Tukey's HSD test across phases in Cluster 3 ContL

| group 1 | group 2 | diff | 95% CI | p | Cohen's d |
|---|---|---|---|---|---|
| B | P1 | 0.556 | [-2.204, 3.316] | 0.983 | 0.276 |
| B | P2 | 1.896 | [-0.864, 4.656] | 0.179 | 0.773 |

| | | | | | |
|---|---|---|---|---|---|
| B | P3 | 2.554 | [-0.206, 5.314] | 0.022 | 1.014 |
| B | P4 | 2.565 | [-0.195, 5.324] | 0.021 | 0.974 |
| B | P5 | 2.797 | [0.038, 5.557] | **0.009** | 0.94 |
| P1 | P2 | 1.34 | [-1.420, 4.100] | 0.558 | 0.503 |
| P1 | P3 | 1.998 | [-0.762, 4.758] | 0.136 | 0.733 |
| P1 | P4 | 2.009 | [-0.751, 4.768] | 0.132 | 0.709 |
| P1 | P5 | 2.241 | [-0.518, 5.001] | 0.066 | 0.71 |
| P2 | P3 | 0.658 | [-2.102, 3.418] | 0.964 | 0.215 |
| P2 | P4 | 0.669 | [-2.091, 3.428] | 0.962 | 0.212 |
| P2 | P5 | 0.901 | [-1.859, 3.661] | 0.873 | 0.261 |
| P3 | P4 | 0.011 | [-2.749, 2.770] | 1 | 0.003 |
| P3 | P5 | 0.243 | [-2.516, 3.003] | 1 | 0.07 |
| P4 | P5 | 0.233 | [-2.527, 2.992] | 1 | 0.065 |

**Supplementary Table 42**

Tukey's HSD test across phases in Cluster 4 ContL

| group 1 | group 2 | diff | 95% CI | p | Cohen's d |
|---|---|---|---|---|---|
| B | P1 | 0.515 | [-3.552, 4.582] | 0.998 | 0.42 |
| B | P2 | 1.468 | [-2.600, 5.535] | 0.789 | 0.753 |
| B | P3 | 1.287 | [-2.78, 5.355] | 0.866 | 0.567 |
| B | P4 | 2.587 | [-1.480, 6.654] | 0.225 | 1.157 |
| B | P5 | 2.591 | [-1.477, 6.658] | 0.224 | 1.221 |
| P1 | P2 | 0.953 | [-3.115, 5.020] | 0.959 | 0.472 |
| P1 | P3 | 0.772 | [-3.295, 4.840] | 0.984 | 0.331 |
| P1 | P4 | 2.072 | [-1.995, 6.139] | 0.463 | 0.902 |
| P1 | P5 | 2.076 | [-1.992, 6.143] | 0.461 | 0.949 |
| P2 | P3 | -0.181 | [-4.248, 3.887] | 1 | 0.065 |
| P2 | P4 | 1.119 | [-2.948, 5.187] | 0.921 | 0.407 |
| P2 | P5 | 1.123 | [-2.945, 5.190] | 0.92 | 0.422 |
| P3 | P4 | 1.3 | [-2.767, 5.367] | 0.861 | 0.435 |
| P3 | P5 | 1.303 | [-2.764, 5.371] | 0.86 | 0.449 |
| P4 | P5 | 0.003 | [-4.064, 4.071] | 1 | 0.001 |

**Supplementary Table 43**

Tukey's HSD test across clusters in Baseline ContL

| group 1 | group 2 | diff | 95% CI | p | Cohen's d |
|---|---|---|---|---|---|
| C1 | C2 | -0.703 | [-1.869, 0.464] | 0.231 | 0.32 |
| C1 | C3 | -1.667 | [-3.099, -0.235] | **0.002** | 0.778 |
| C1 | C4 | -2.584 | [-4.885, 0.282] | **0.003** | 1.188 |
| C2 | C3 | -0.964 | [-2.562, 0.634] | 0.23 | 0.49 |
| C2 | C4 | -1.881 | [-4.289, 0.527] | 0.069 | 0.949 |
| C3 | C4 | -0.917 | [-3.464, 1.630] | 0.668 | 0.573 |

**Supplementary Table 44**

Tukey's HSD test across clusters in Play 1 ContL (not conducted)

| group 1 | group 2 | diff | 95% CI | p | Cohen's d |
|---|---|---|---|---|---|
| C1 | C2 | | | | |
| C1 | C3 | | | | |
| C1 | C4 | | | | |
| C2 | C3 | | | | |
| C2 | C4 | | | | |
| C3 | C4 | | | | |

**Supplementary Table 45**

Tukey's HSD test across clusters in Play 2 ContL (not conducted)

| group 1 | group 2 | diff | 95% CI | p | Cohen's d |
|---|---|---|---|---|---|
| C1 | C2 | | | | |
| C1 | C3 | | | | |
| C1 | C4 | | | | |
| C2 | C3 | | | | |
| C2 | C4 | | | | |
| C3 | C4 | | | | |

**Supplementary Table 46**

Tukey's HSD test across clusters in Play 3 ContL

| group 1 | group 2 | diff | 95% CI | p | Cohen's d |
|---|---|---|---|---|---|
| C1 | C2 | 0.384 | [-0.920, 1.689] | 0.789 | 0.18 |
| C1 | C3 | 1.853 | [0.252, 3.455] | **0.002** | 0.782 |
| C1 | C4 | -0.33 | [-2.903, 2.243] | 0.978 | 0.149 |
| C2 | C3 | 1.469 | [-0.318, 3.256] | 0.05 | 0.581 |
| C2 | C4 | -0.714 | [-3.406, 1.978] | 0.836 | 0.313 |
| C3 | C4 | -2.184 | [-5.031, 0.664] | 0.077 | 0.706 |

**Supplementary Table 47**

Tukey's HSD test across clusters in Play4 ContL

| group 1 | group 2 | diff | 95% CI | p | Cohen's d |
|---|---|---|---|---|---|
| C1 | C2 | 0.488 | [-0.817, 1.793] | 0.64 | 0.234 |
| C1 | C3 | 1.725 | [0.124, 3.327] | **0.005** | 0.756 |
| C1 | C4 | 0.831 | [-1.742, 3.405] | 0.738 | 0.41 |
| C2 | C3 | 1.238 | [-0.550, 3.025] | 0.131 | 0.449 |
| C2 | C4 | 0.343 | [-2.350, 3.036] | 0.978 | 0.138 |
| C3 | C4 | -0.894 | [-3.743, 1.955] | 0.755 | 0.277 |

**Supplementary Table 48**

Tukey's HSD test across clusters in Play 5 ContL (not conducted)

| group 1 | group 2 | diff | 95% CI | p | Cohen's d |
|---|---|---|---|---|---|
| C1 | C2 | | | | |
| C1 | C3 | | | | |

| | |
|---|---|
| C1 | C4 |
| C2 | C3 |
| C2 | C4 |
| C3 | C4 |
